# The miR-29 family facilitates the activation of NK-cell immune responses by targeting the B7-H3 immune checkpoint in neuroblastoma

**DOI:** 10.1038/s41419-024-06791-7

**Published:** 2024-06-18

**Authors:** Anup S. Pathania, Haritha Chava, Nagendra K. Chaturvedi, Srinivas Chava, Siddappa N. Byrareddy, Don W. Coulter, Kishore B. Challagundla

**Affiliations:** 1 Department of Biochemistry and Molecular Biology & The Fred and Pamela Buffett Cancer Center, University of Nebraska Medical Center, Omaha, NE 68198 USA; 2 Department of Pharmacology and Experimental Neuroscience, University of Nebraska Medical Center, Omaha, NE 68198 USA; 3 Department of Pediatrics, Division of Hematology/Oncology, University of Nebraska Medical Center, Omaha, NE 68198 USA; 4 The Child Health Research Institute, University of Nebraska Medical Center, Omaha, NE 68198 USA

**Keywords:** Paediatric cancer, Outcomes research

## Abstract

Neuroblastoma (NB) is a highly aggressive pediatric cancer that originates from immature nerve cells, presenting significant treatment challenges due to therapy resistance. Despite intensive treatment, approximately 50% of high-risk NB cases exhibit therapy resistance or experience relapse, resulting in poor outcomes often associated with tumor immune evasion. B7-H3 is an immune checkpoint protein known to inhibit immune responses. MicroRNAs (miRNAs) are small non-coding RNAs involved in post-transcriptional gene regulation. Our study aims to explore the impact of miRNAs on B7-H3 regulation, the anti-tumor immune response, and tumorigenicity in NB. Analysis of NB patients and patient-derived xenograft tumors revealed a correlation between higher B7-H3 expression and poorer patient survival. Notably, deceased patients exhibited a depletion of miR-29 family members (miR-29a, miR-29b, and miR-29c), which displayed an inverse association with B7-H3 expression in NB patients. Overexpression and knockdown experiments demonstrated that these miRNAs degrade B7-H3 mRNA, resulting in enhanced NK cell activation and cytotoxicity. In vivo, experiments provided further evidence that miR-29 family members reduce tumorigenicity, macrophage infiltration, and microvessel density, promote infiltration and activation of NK cells, and induce tumor cell apoptosis. These findings offer a rationale for developing more effective combination treatments that leverage miRNAs to target B7-H3 in NB patients.

## Introduction

NB is the most common pediatric cancer diagnosed in children under the age of five and accounts for about 15% of childhood cancer-related deaths [1]. Treatment strategies for NB patients depend on the risk group they fall into, which includes low-risk, intermediate-risk, and high-risk categories. Unfortunately, approximately 50% of children with high-risk NB experience more aggressive tumor relapse, resulting in less than a 20% five-year overall survival rate [2–5]. High-risk NB patients pose significant challenges as they require intensive chemotherapy and radiation therapy, and their chances of tumor regression are lower compared to other risk groups [6–8]. Moreover, the extensive treatment regimens used in high-risk NB patients have substantial adverse effects on their overall well-being and quality of life [9]. Natural killer (NK) cell activity-dependent immunotherapy using Dinutuximab, an antibody directed against GD_2_, a disialoganglioside expressed on the surface of NB cells in combination with chemotherapy has shown survival benefits in high-risk NB patients. However, a significant proportion of patients still relapse and fail to respond due to various mechanisms that tumors employ to develop therapy resistance and suppress anti-tumor immune responses [10, 11].

B7-H3 (coded by gene CD276) is a transmembrane protein that belongs to the B7 family of immune checkpoint proteins. It plays a role in inhibiting both adaptive and innate immune responses [12–14]. High levels of B7-H3 expression have been detected in high-grade solid tumors compared to corresponding normal healthy cells [13, 15]. This overexpression is associated with an increased risk of disease progression, as B7-H3 helps the tumor cells evade immune detection and supports tumor growth by inhibiting functional T and NK cell activation [15–17]. In preclinical studies, various strategies have been explored to target B7-H3, with some showing promising responses, making it a potential target for intervention [18, 19]. Despite the promising results in preclinical models, some of these challenges include low surface antigen expression on tumor cells, antigen heterogeneity, limited tumor penetration by Chimeric Antigen Receptor T*-*cells (CAR-T), and severe toxicities associated with the treatment. In clinical trials, GD2-CAR-T cell therapy for children with relapsed or refractory NB was well tolerated without off-tumor effects, but it failed to achieve objective clinical responses [20]. This highlights the need for developing novel approaches that target the upstream regulatory pathway of B7H3 to overcome the current challenges in NB immunotherapy.

MiRNAs are small non-coding RNAs that play a crucial role in post-transcriptional gene regulation. They are typically 18–25 nucleotides in length and can bind to the 3′-untranslated region (3′-UTR) of target genes, leading to the repression of gene expression [21]. In NB, miRNAs have been implicated in regulating signaling pathways involved in growth, progression, and immune response [22–25]. The miR-29 family, consisting of miR-29a, miR-29b, and miR-29c, has been previously shown to act as tumor suppressors [26]. Our previous research has shown that NB cells transfer miR-21 in exosomes to surrounding monocytes, triggering the induction of oncogenic miR-155 expression through exosomes, which is involved in inducing chemotherapy resistance [27]. Considering that immunotherapy is often combined with chemotherapy, understanding the role of miRNAs in regulating immune cell function and survival in NB patients becomes crucial.

The present manuscript aims to investigate the clinical significance of B7H3 and the miR-29 family (miR-29a, miR-29b, and miR-29c) in NB patients and PDX tumors, with a focus on understanding the regulatory role of these miRNAs on B7H3 expression, immune cell function, NB growth, and tumorigenicity using a combination of in vitro and in vivo approaches.

## Results

### Patients with higher B7H3 expression exhibited poor survival in patients

**Fig. 1:**
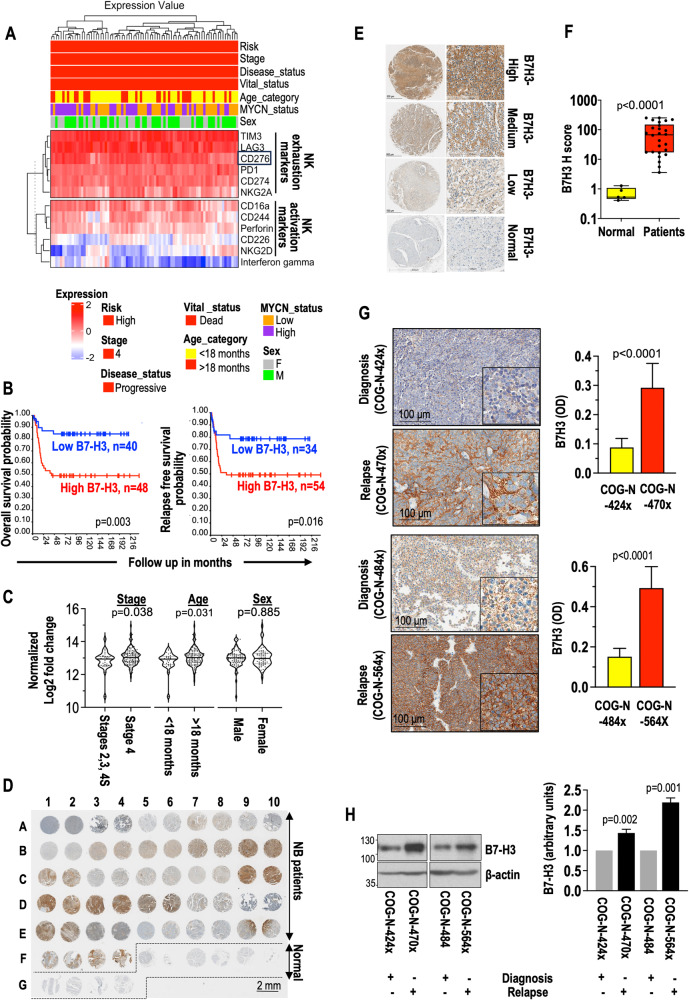
Elevated B7-H3 expression is associated with high-risk, stage 4 disease progression, poor survival, and an increased likelihood of relapse in NB patients. **A** The heatmap illustrates the differential co-expression of genes associated with NK-cell exhaustion and activation within the NB patient dataset (GSE62564, n = 498). The heatmap was generated using log-transformed and scaled expression values. **B** The Kaplan–Meier curves illustrate the association between different levels of B7-H3 expression and both overall survival (left) and relapse-free survival (right) in a cohort of GSE16476 (n = 88) dataset. Patients with elevated B7-H3 expression exhibited shorter survival outcomes. **C** The violin plot illustrates the expression of B7-H3 in NB patients from the TARGET (n = 151) dataset. **D** IHC images show B7-H3 staining in NB patient tumor samples (n = 27, A1-F4 in duplicates) and normal peripheral nerve tissue microarrays (n = 10, F5-G4 in duplicates). Scale bar: 2 mm. **E** The signal intensity of B7-H3 was further divided into higher, medium, and low and compared with normal peripheral nerve tissue samples. Scale bar: 100 µm. **F** The intensity of B7-H3 staining is quantified using an H-score system, and comparisons were made between NB patient samples and normal peripheral nerve tissue samples. *P < 0.0001, Mann–Whitney U-test. **G** IHC images of surface B7-H3 staining in two different PDX tumor samples from NB patients at the diagnosis and relapse stages. Magnified images were shown in the insets. Scale bar: 100 *μ*m. The color intensity of B7-H3 staining was quantified and presented as optical density (OD) units. **H** Western blotting analysis of B7-H3 protein levels in whole lysates of two different PDX tumors from NB patients at the diagnosis and relapse stages.

NK cells are the main effector cells of the innate immune system in NB. We first tested the status of NK activation and exhaustion markers in high-risk NB patients using co-expression analysis from the RNA sequencing data of GSE62564 (n = 498) dataset. As depicted in Fig. 1A, we noticed the trend of higher expressing NK exhaustion genes while decreasing NK activation genes. We conducted survival probability analysis (GSE16476, n = 88) [28] using NK exhaustion genes and observed a significant association between higher B7H3 expression and poor overall and event-free survival in high-risk patients (Fig. 1B). This association was consistently observed in two independent datasets: GSE85047 (n = 283) [29] and GSE62564 (n = 498) (Supplementary Fig. S1A). Additionally, our findings were supported by the observation of higher B7-H3 expression in high-risk (GSE62564, n = 498, Supplementary Fig. S1B) and stage 4 NB patients (TARGET, n = 151, Fig. 1C; GSE45547, n = 649 [30], Supplementary Fig. S1C), as well as in patients with higher progression and deceased outcomes in the GSE16476 (n = 88) [28] and GSE62564 (n = 498) datasets (Supplementary Fig. S1D). Furthermore, we noted elevated B7-H3 expression in patients with unfavorable histology as well as those with a higher Mitosis-Karyorrhexis Index (MKI), which is indicative of poor prognosis, in comparison to their respective control patients (TARGET) (Supplementary Fig. S1E, F).

Next, we performed B7H3 immunohistochemistry (IHC) staining in NB patient tumors and PDX tumors at diagnosis and relapse stages. We utilized a tissue microarray (TMA) slide that contains 26 histological NB patient tumors representing different NB stages and five corresponding normal peripheral nerve tissues in duplicates. Patient tumors showed strong B7H3 staining indicated by intense brown staining, compared to normal peripheral nerve tissues (Fig. 1D). Based on B7H3 signal intensity, we divided tumors into higher, medium, and lower expressions and compared them with normal nerve tissues (Fig. 1E). The quantification of B7H3 expression was performed using the HALO image analysis platform and presented as H-score. The H-score analysis showed that patient tumors showed over 10-100-fold higher expression of B7H3 compared to non-neoplastic peripheral nerve tissues (Fig. 1F). Next, we examined B7H3 expression in PDX tumors collected at diagnosis and relapsed stages through IHC, Western blotting, and flow cytometry analyses. We isolated human NB cells by flow analysis from PDX tumors freshly using GD2 staining, as they express higher GD2. As depicted in Fig. 1G, H and Supplementary Fig. S1G, a trend of B7H3 up-regulation was seen in relapse-specific PDX tumors compared to diagnosis. These analyses demonstrate that B7H3 is exhibited elevated expression in relapse and is associated with poor survival in NB patients.

### Higher miR-29a, miR-29b, and miR-29c levels were associated with better survival in NB patients

**Fig. 2:**
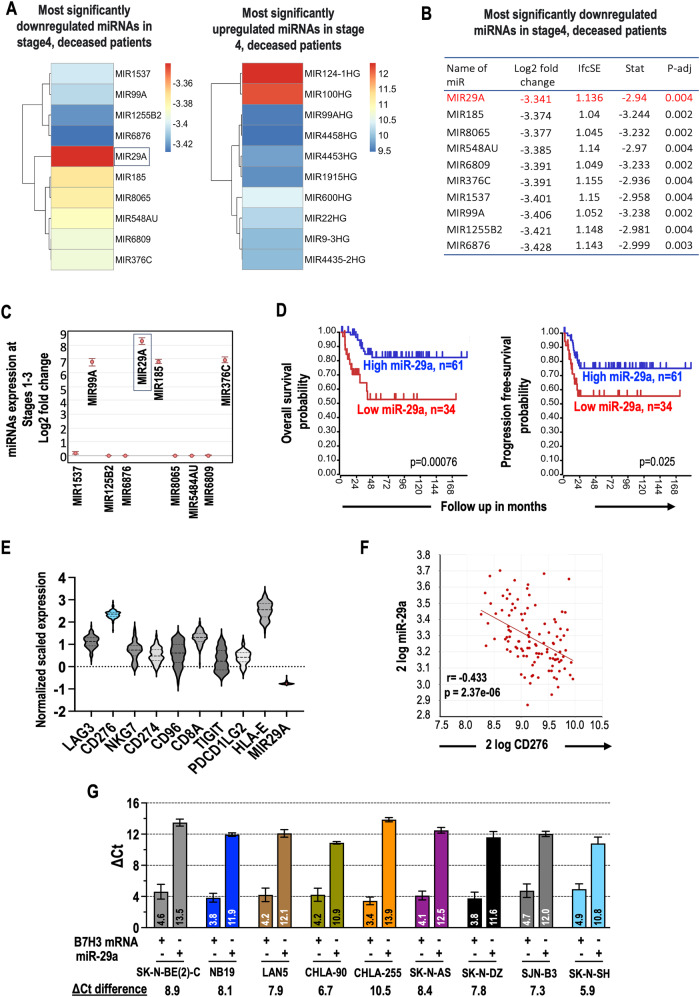
High-risk NB patients tend to show decreased expression of miR-29a, miR-29b, and miR-29c, together with poor clinical outcome in NB patients. **A** The heatmap displays miRs that are highly significant (adjusted p-value < 0.005) and are among the top-ranked upregulated and downregulated miRs in deceased patients from high-risk stage 4 NB patient cohorts. This analysis was conducted on the log-transformed TARGET dataset. **B** The log2fold change values of significant (adjusted p-value < 0.005), top-ranked, downregulated miRs in deceased patients from high-risk, stage 4 NB patient cohort (TARGET, n = 151). lfcSE = log 2-fold change standard error; stat = Wald statistic (larger absolute values of the stat indicate greater evidence of differential expression); padj = p adjusted values. **C** The log2-fold increase in the expression levels of miR-29a in low risk NB patients derived from the GSE155945 (n = 97) dataset. Notably, miR-29a showed higher expression levels in low or intermediate-risk stages compared to other miRs. **D** Kaplan–Meier curves display the association between varying levels of miR-29a and overall survival (left) and progression-free survival (right) in NB patients from the GSE155945 (n = 97) dataset. Patients with elevated miR-29a expression showed improved survival outcomes. **E**, **F** Graphs illustrate the inverse correlation between miR-29a and B7-H3 expression in NB patients, derived from the TARGET and Exp NB (TAE-684/shALK) - de Preter - 113 - RMA - u133p2 (n = 113) datasets. **G** The graph illustrates the physiological expression levels of B7-H3 mRNA and miR-29a in nine distinct NB cell lines. These results are presented as the mean ± standard error (SEM) and are derived from 3–4 independent biological experiments.

MiRNAs or miRs are involved in post-transcriptional gene regulation. We investigated the association between miRs and patient survival using the TARGET (n = 151) NB patient dataset. Significant downregulated and upregulated miRs were identified (Fig. 2A), and miR-29a was the most significantly downregulated in stage 4 patients (Fig. 2B). This finding was validated in an independent cohort of NB patients (GSE155945, n = 97), where miR-29a showed higher expression in low or intermediate-risk stages (Fig. 2C and Supplementary Fig. S2A). Kaplan–Meier survival analysis indicated that higher levels of miR-29a, miR-29b, and miR-29c were associated with better overall and progression-free survival (Fig. 2D and Supplementary Fig. S2B, C). In metastatic NB patients (GSE94035, n = 86) [31], disseminated tumor cells (DTCs) in the bone marrow (BM) had lower miR-29a levels compared to normal BM-derived mononuclear cells (MNCs) at diagnosis and relapse stages. Conversely, DTCs exhibited significantly higher B7-H3 levels than BM-MNCs at diagnosis and relapse stages, and primary tumors in these patients also showed downregulated miR-29a and upregulated B7-H3 (Supplementary Fig. S2D). Furthermore, a negative correlation between miR-29a and B7-H3 was observed in an independent RNA Seq dataset of NB patients (TARGET, n = 151 and De Preter −113 [32]), indicating an inverse relationship (r = −0.433, p = 2.37e-6) (Fig. 2E, F). In addition, basal B7H3 mRNA and miR-29 expressions were examined in a panel of nine NB cell lines through qRT-PCR, and the delta Ct values demonstrated higher B7-H3 expression and lower levels of miR-29s in all tested NB cell lines (Fig. 2G and Supplementary Fig. S2E). The cell lines SK-N-B(E)2-C, SK-N-AS, and CHLA-255 with the highest Ct value differences between B7H3 mRNA and miR-29s, were selected for further investigation of the regulatory aspects of B7H3 by miRs.

### MiR-29a, miR-29b, and miR-29c target B7H3 in NB cells

**Fig. 3:**
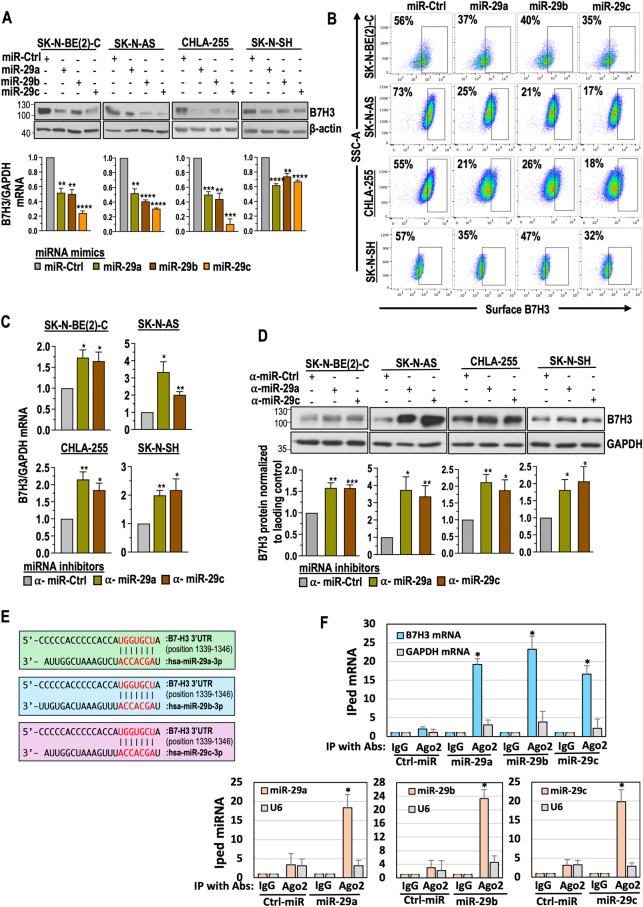
miR-29a, miR-29b, and miR-29c target B7-H3 in NB cells. **A** Western blot (upper panel) analysis showing B7-H3 total protein levels in NB cells transfected with miR-29a, miR-29b, and miR-29c, or miR Ctrl mimics for 48 h. Quantification graph (lower panel) showing B7-H3 mRNA levels in NB cells transfected with miR-29a, miR-29b, and miR-29c, or miR Ctrl mimics for 48 h, measured using qRT-PCR. **B** Representative flow cytometric plots showing B7-H3 surface expression in SK-N-BE(2)-C, SK-N-AS, CHLA-255 and SK-N-SH cells transfected with miR-29a, miR-29b, and miR-29c, or miR Ctrl mimics for 48 h. RT-qPCR (**C**) and Western blot analysis (**D**) demonstrating B7-H3 mRNA and protein levels in NB cells transfected with inhibitors of miRNAs (labeled with $a$) such as $a$-miR-29a, $a$-miR-29c, and α-Scr Ctrl for 48 h. Densitometric quantification graph representing the relative B7-H3 protein levels normalized to the control group. **E** The sequence alignment shows the predicted binding sites between miR-29a, miR-29b, and miR-29c and 3’UTR of B7-H3 mRNA. Complementary sequences of B7-H3 mRNA and miRs are shown red. **F** A RT-qPCR quantification graph for Ago2-occupied B7-H3 mRNA (upper panel) and Ago2-occupied miR-29a, miR-29b, or miR-29c (lower panel) is shown. SK-N-BE(2) cells were transfected with miR-29a, miR-29b, miR-29c, or control miRs for 48 h followed by IP with an anti-Ago2 antibody. Ago2-bound RNA complexes were eluted, purified, and quantified for Ago2-bound mRNA and miRs by RT-qPCR using TaqMan assays. GAPDH mRNA and U6, a non-coding small nuclear RNA, were used as non-specific controls. The data were compared with the control IgG-bound mRNA or miR and set to one for normalization. Data are presented as mean ± standard error from three to four independent biological replicates. Statistical analysis was performed using a two-sided unpaired t-test. *p < 0.05, **p < 0.01, ***p < 0.001, and ****p < 0.0001.

We first screened B7H3 levels in nine NB cell lines and selected SK-N-B(E)2-C, SK-N-AS, CHLA-255, and SK-N-SH for further investigation (Supplementary Fig. S3A). To investigate the impact of miR-29a, miR-29b, and miR-29c on B7-H3 regulation, we transfected the NB cell lines SK-N-BE(2)-C, SK-N-AS, CHLA-255 and SK-N-SH with miR-29a, miR-29b, miR-29c, and control mimics. The fold change increase in miR-29a and miR-29c after miRNA transfection is depicted in Supplementary Fig. S3B. As for miR-29b, we presented delta Ct values post miRNA transfection due to the undetectable basal levels in NB cell lines (Supplementary Fig. S3C). NB cells transfected with miR-29 mimics for 48 h, resulting in a significant depletion of B7H3 mRNA and protein (Fig. 3A and Supplementary Fig. S3D). Stable miR expression in NB cells yielded similar results (Supplementary Fig. S3E). Flow cytometry confirmed reduced B7-H3 surface expression in NB cells upon miR-29a, miR-29b, and miR-29c transfection (Fig. 3B and Supplementary Fig. S3F, G). Silencing endogenous miRs using inhibitors led to increased B7H3 mRNA and protein expressions (Fig. 3C, D and Supplementary Fig. S3H).

**Fig. 4:**
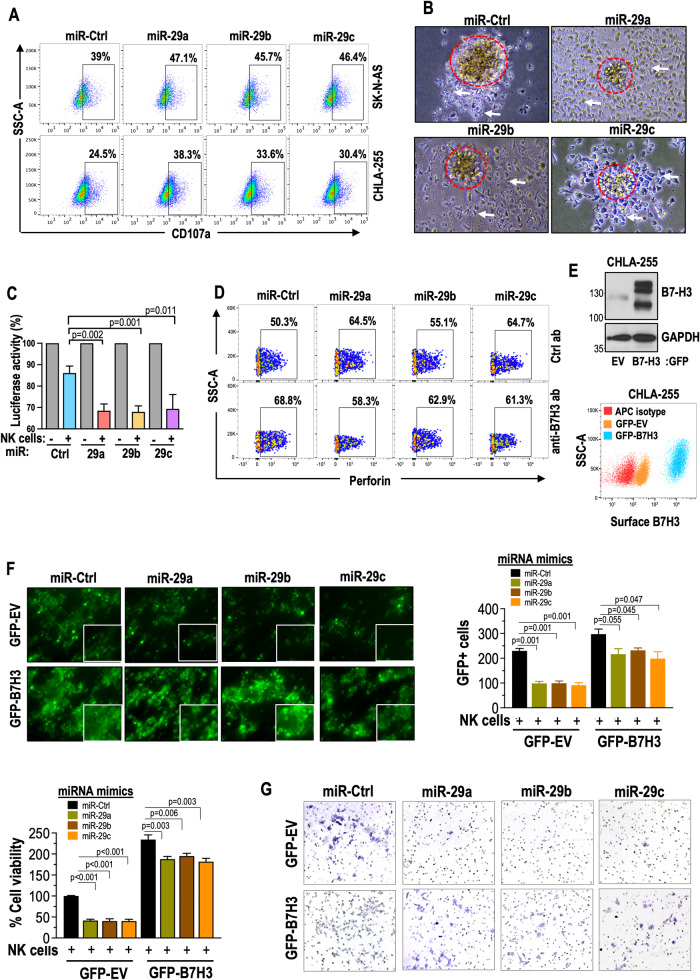
miR-29a, miR-29b, and miR-29c enhance NK cell activation and NK-mediated cytotoxicity in NB cells. **A** Representative flow cytometry plots illustrate the expression of CD107 in NK cells co-cultured (E:T = 1:2 for 5 h) with NB cells that were transfected with miR-29a, miR-29b, miR-29c, or miR Ctrl mimics for 48 h. The percentage of CD107 + NK cells is indicated in the plots. **B** Phase-contrast microscopy images of SK-N-B(E)2-C spheroids stably expressing miR-29a, miR-29b, and miR-29c, co-cultured (E:T = 2:1) with IL-15-treated NK cells for 48 h. The dotted circle represents cancer cell spheroids, whereas the white arrows indicate NK cells. **C** A quantification graph demonstrates normalized luciferase activity in SK-N-BE(2)-luciferase cells stably expressing miR-29a, miR-29b, and miR-29c upon co-culture (E:T = 1:1) with activated NK cells for 5 h. **D** Representative flow cytometry plots show the expression of perforin in NK cells co-cultured (E:T = 1:2 for 5 h) with NB cells pretreated with an anti-B7-H3 antibody (5 μg) for 24 h and transfected with miR-29a, miR-29b, miR-29c, or miR Ctrl mimics for an additional 24 h. The percentage of perforin+ NK cells is indicated in the plots. **E** Western blotting and flow cytometric expression of total and surface B7-H3 in CHLA-255 cells stably expressing GFP EV or GFP-B7-H3. **F** GFP-based digital fluorescence microscopy assay displaying GFP fluorescence in CHLA-255 cells, which stably express either GFP-B7H3 or a GFP-EV control. These cells were transfected with miR-29a, miR-29b, or miR-29c for 48 h and then co-cultured with activated NK cells (E:T = 1:2) for an additional 5 h. Quantification of GFP fluorescence is shown in the graphs on the right. After removing the NK cells, the CHLA-255 cells were allowed to grow for another 48 h before performing a cell viability assay. Live cells, identified as trypan blue negative, were manually counted, and the percentage of cell viability was calculated using GFP-EV cells as control (100%). **G** Transwell cell invasion assay images demonstrate the invasion ability of CHLA-255 cells stably expressing GFP-B7H3 or GFP-EV control, transfected with miR-29a, miR-29b, and miR-29c for 24–48 h. These cells were co-cultured (E:T = 1:2) with activated NK cells for an additional 5 h. After incubation, NK cells were removed from the medium, and NB cells were allowed to grow for an additional 24 h. The cells were counted, and 15,000 live cells were seeded into Corning Transwell polycarbonate membrane cell culture inserts coated with Matrigel for 72 h. The cells that migrated to the bottom chamber were stained using crystal violet and quantified. The data are presented as mean ± SEM from 3–5 independent experiments. P-values were determined using a two-sided unpaired t-test.

Next, we used TargetScan to predict the interaction between the 3’UTR of B7-H3 and miR-29a, miR-29b, and miR-29c (Fig. 4E). Subsequently, Ago2 immunoprecipitation (IP) was performed to assess their binding to B7-H3 mRNA in NB cells. Ago2, a core component of the RNA-induced silencing complex (RISC), guides miRNAs to their target mRNAs, indicating gene regulation by specific miRNAs. [33, 34]. In Fig. 3F, anti-Ago2 antibodies successfully immunoprecipitated B7-H3 mRNA, but not GAPDH mRNA. Ago2 also immunoprecipitated miR-29a, miR-29b, and miR-29c, but not U6, a small nucleolar RNA. These findings suggest that miR-29a, miR-29b, and miR-29c directly regulate B7-H3 through interaction with its mRNA in NB cells. Collectively, these results demonstrated that miR-29a, miR-29b, and miR-29c target B7H3 in NB cells.

### miR-29a, miR-29b, and miR-29c promote NK cell activation and NK-mediated cytotoxicity by targeting B7-H3 in NB cells

B7-H3 expression on the tumor cell surface is primarily involved in immune cell dysfunctions, and recent studies have reported its prognostic value in different cancer types [35]. B7-H3 overexpression on tumor cells is known to attenuate NK-cell activation and functions [14]. We investigated the impact of miR-29a, miR-29b, and miR-29c on NK cell-mediated anti-tumor immune responses against NB cells. NB cells (SK-N-AS and CHLA-255) were transfected with miR-29a, miR-29b, miR-29c, or miR-Ctrl for 48 h and then co-cultured with activated human NK cells for an additional 5 h at an effector-to-target (E:T = 1:2) ratio. NK cells were isolated from healthy donors and expanded with IL-2 and irradiated K562 CSTX002 mbIL21.41bbL cells. The purity of NK cells (CD3-CD56 + CD16+) was checked by immunophenotyping using flow cytometry (supplementary Fig. S4A–C). Following co-culture, NK cell activation markers, including CD107a, were assessed. Elevated CD107a and perforin expressions were observed in NK cells co-cultured with miR-expressing SK-N-AS and CHLA-255 cells, indicating increased NK cell activation compared to control miRs (Fig. 4A and supplementary Fig. S4D–H).

Additionally, we treated SK-N-B(E)2-C spheroids, stably expressing miR-29a, miR-29b, miR-29c, or miR-Ctrl, with IL-15-treated NK cells for 48 h (E:T = 2:1) and monitored spheroid integrity by measuring their volume using picture-based measurements. Spheroids expressing miR-29a/b/c showed smaller and fragmented structures compared to miR-Ctrl spheroids at 48 h, indicating that miR-29a/b/c promoted NK-mediated disruption of the tumor structure (Fig. 4B, Supplementary Fig. S4I). Furthermore, we assessed the cytotoxicity of NK cells against NB cells using SK-N-BE(2)-luciferase cells stably expressing the luciferase gene. These cells were transfected with miR-29a, miR-29b, miR-29c, or miR-Ctrl mimics for 48 h and then co-cultured with activated NK cells (E:T = 1:1) for 5 h, followed by luciferase analysis. Cells expressing miRs exhibited lower luciferase activity, indicating increased NB cell death upon NK cell cytotoxicity (Fig. 4C). There was a decrease in TNFα but an increase in IL-2 secretion in cells expressing miR-29s compared to control cells upon co-culture with activated NK cells. (Supplementary Fig. S4J). Overall, these results demonstrate that miR-29a, miR-29b, and miR-29c enhance NK cell activation and NK-mediated cytotoxicity in NB cells.

To test the influence of B7-H3 on the impact of miR-29a, miR-29b, and miR-29c on NK cell activation and functions in NB. We first transfected SK-N-AS cells with miR-29a, miR-29b, and miR-29c mimics, followed by blocking with anti-B7-H3 or IgG control antibodies for an additional 24 h. These cells were co-cultured (E:T = 1:2) with NK cells for 5 h, followed by perforin analysis. NK cells showed enhanced perforin secretion upon co-culture with miR-treated NB cells, but this effect was not affected by B7-H3 antibody treatment (Fig. 4D, supplementary Fig. S4K). Secondly, we used CHLA-255 cells that stably express GFP-B7-H3 or GFP-EV. B7-H3-overexpressing cells exhibited higher B7-H3 expression (Fig. 4E). These cells were transiently transfected with miR-29a, miR-29b, and miR-29c, cocultured with NK cells and NB cell viability was assessed. In the presence of NK cells and miR-29a/b/c treatment, NB cells showed a significant reduction of cell viability, but this reduction was smaller in B7-H3-overexpressing cells (Fig. 4F). Lastly, we examined NB cell invasiveness in vitro using a Matrigel assay. CHLA-255 cells showed reduced invasion upon NK cells and miR-29a/b/c treatment. However, B7-H3 overexpression in these cells led to increased invasion even in miR-treated cells, suggesting that B7-H3 impacts miR-29a/b/c-promoted inhibition of invasion in the presence of NK cells (Fig. 4G).

### miR-29a, miR-29b, and miR-29c regulate NK cell-mediated anti-tumor immune response in NB xenografts

**Fig. 5:**
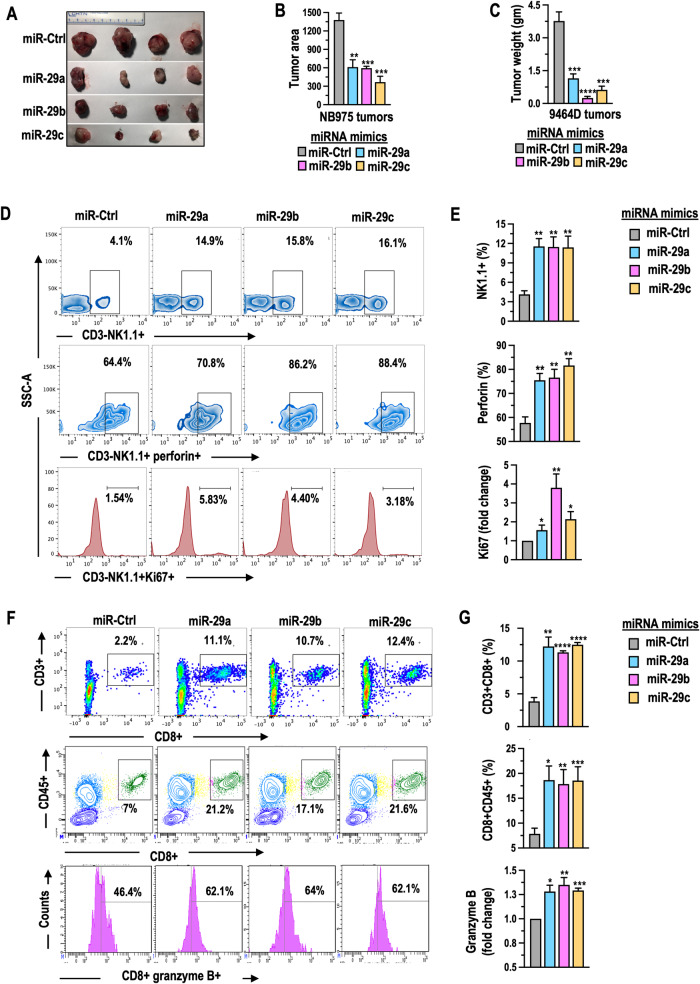
miR-29a, miR-29b, and miR-29c promote NK and T cell-mediated antitumor immune responses in NB xenografts. Photographs displaying tumor images (**A**), graphs illustrating the quantification of average tumor area, tumor weight (**B**, **C**) in C57BL/6 mice that received subcutaneous injections of murine 9464D cells (n = 5) or NB-975 cells (n = 4) expressing GFP-miR-29a, miR-29b, miR-29c, or GFP-control (ctrl) miRs. **D** Representative flow cytometry plots showing NK cell infiltration and the expression of perforin and Ki67 in NK cells (CD3-NK1.1 + ) gated on CD45+ cells, as analyzed from single-cell suspensions of tumor tissues obtained from C57BL/6 mice that received a single injection of murine 9464D cells expressing GFP-miR-29a, miR-29b, miR-29c, or GFP-control (ctrl) miRs and were observed for 30 days. **E** Quantification graphs corresponding to (**D**). **F** Representative flow cytometry plots demonstrating CD8 + T cell infiltration and the expression of granzyme in CD8+ cells (CD3 + CD8 + ) gated on CD45+ cells, as analyzed from single-cell suspensions of tumor tissues obtained from C57BL/6 mice that received a single injection of murine 9464D (upper panel) or NB975 (lower panels) cells expressing GFP-miR-29a, miR-29b, miR-29c, or GFP-control (ctrl) miRs and were observed for 30 days. **G** Quantification graphs corresponding to (**F**).

We investigated the impact of miR-29a/b/c on antitumor immunity in immune-competent C57/BL6 mice, using murine NB cell lines 9464D and NB975. Female mice received subcutaneous injections of these cells, and after 30 days, we assessed tumor growth and the infiltration of cytotoxic NK and T cells. (i) tumor growth: As displayed in Fig. 5A–C, tumors in mice with miR-29a/b/c-expressing 9464D or NB975 cells showed significant size and weight reduction compared to control tumors. (ii) NK Cell activation and recruitment: As displayed in Fig. 5D, E and Supplementary Fig. S5A, the infiltrated NK cell population (CD3-NK1.1+) increased with a higher percentage of perforin expressing NK cells (CD3-NK1.1+Perforin+) and elevated Ki67 expression in NK cells (CD3-NK1.1 + Ki67+) in miR-29-expressing tumors. Further IHC staining of CD161c/NK1.1, another NK cell infiltration marker exhibited a strong positive signal in tumors of miRs expression compared to control miRs (Supplementary Fig. S5B). (iii) CD8 + T cell infiltration: As depicted in Fig. 5F, G and Supplementary Fig. S5C, the percentage of CD3 + CD8+ cells and CD45 + CD8+ cells, along with CD8+granzyme+ cells were found to be higher in tumors of miRs compared to tumors of control miRs. These results suggest that miR-29a/b/c promote NK cell activation, recruitment, and enhance CD8 + T cell infiltration into NB tumors, ultimately inhibiting tumor growth.

### miR-29a, miR-29b, and miR-29c increase cleaved caspase-3 expression and reduce tumor microvessel density and macrophage infiltration in NB tumors

**Fig. 6:**
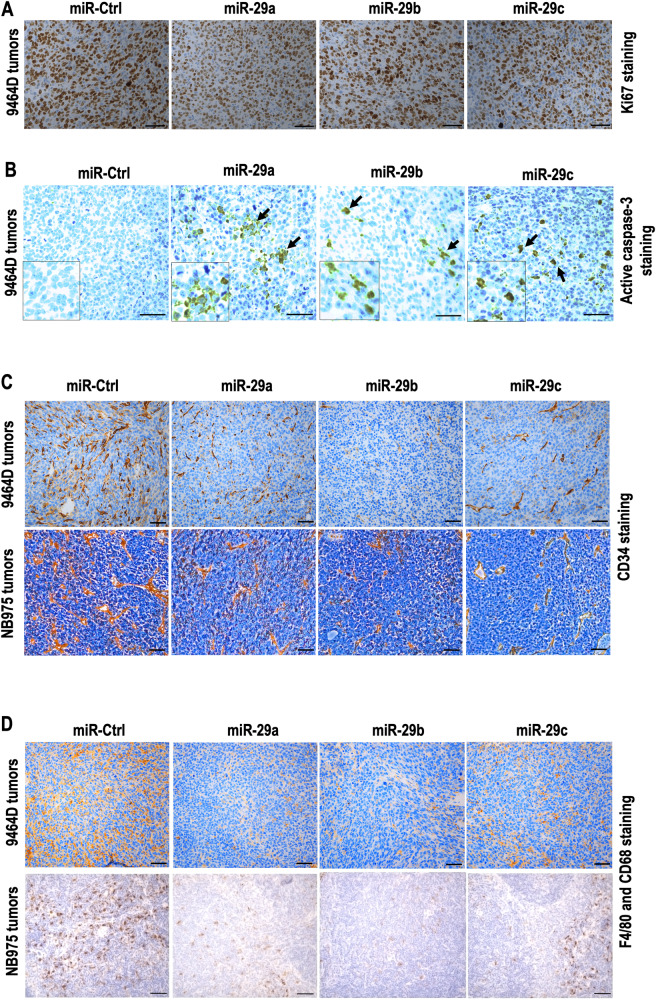
miR-29a, miR-29b, and miR-29c inhibit tumor proliferation, microvessel density and macrophage infiltration in vivo. Representative IHC images depict the expression of (**A**) Ki67, a marker of proliferation, (**B**) cleaved caspase-3, a marker of apoptosis, (**C**) murine endothelial cell marker CD34, indicating microvessel density and (**D**) F4/80 and CD68, indicating tumor-associated macrophage infiltration in tumors derived from mice subcutaneously injected with 9464D ((**A**, **B**) and upper panels of (**C**, **D**)) and NB975 ((**C**, **D**)—lower panels) cells stably expressing miR-29a, miR-29b, miR-29c, or miR-Ctrl (scale bar = 50 μm).

The IHC staining of mouse tumors showed the following. (i) Proliferation and Death: As shown in Fig. 6A, B and Supplementary Fig. S6A and B, tumors with miRs exhibited decreased Ki67 (proliferation) but increased cleaved caspase-3 (cell death) expression. (ii) Vascularization inhibition: MiR-29a, miR-29b, and miR-29c reduced CD34 (a marker for endothelial cells) staining, indicating vascularization inhibition in NB tumors (Fig. 6C, Supplementary Fig. S6C). (iii) Macrophage inhibition: Macrophage populations (F4/80+ and CD68, markers for macrophages) were significantly reduced in miR-29-expressing tumors (Fig. 6D and Supplementary Fig. S6D). Overall, our results suggest that miRs-mediated immune cell activation led to reduced tumor cell proliferation, increased cell death, inhibited vascularization, and decreased macrophage infiltration in vivo.

### miR-29a, miR-29b, and miR-29c inhibit cell growth, proliferation, colony formation, migration, and neurosphere formation in NB cells

**Fig. 7:**
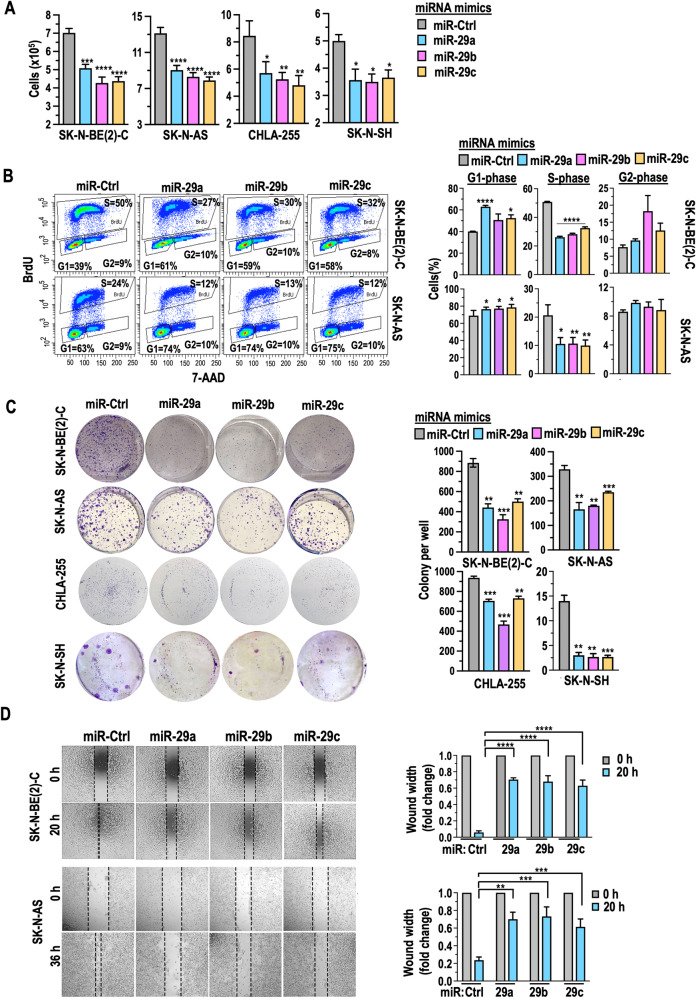
miR-29a, miR-29b, and miR-29c inhibit cell growth, proliferation, colony formation, and migration in NB cells. **A** A quantification graph illustrating cell number, indicating the percentage of viable cells, (**B**) representative flow cytometry plots illustrating the percentage of BrdU-positive cells, indicating cells in the S-phase of the cell cycle, and 7AAD-positive cells indicating cells in G1 and G2 phases, along with a quantification graph displaying the percentage of cells in each phase, (**C**) colony formation images depicting the formation of colonies and quantification graphs accompany the images, showing the number of colonies per well, and (**D**) representative images of wound healing assays demonstrate the closure of a wound over time and quantification graphs accompany the images, illustrating the width of the wound, in NB cells transfected with miR-29a, miR-29b, miR-29c, or a miR-Ctrl for a duration of 48 h. Data points represent the mean ± SEM from three biological replicates. P-values were calculated using two-tailed unpaired t-tests to compare the different experimental groups.

NB cell lines were transfected with miR-29a, miR-29b, miR-29c, or miR-Ctrl mimics for 24–48 h. The following functional assays were performed: (i) Cell number was assessed using the trypan blue exclusion method. As shown in Fig. 7A, cells that received miRs showed a reduction in cell growth. (ii) Cell proliferative capacity was assessed through BrdU uptake by flow cytometry in NB cells. As depicted in Fig. 7B and Supplementary Fig. S7A, BrdU uptake was significantly decreased in NB cells after miRs transfection. Further analysis revealed that NB cells overexpressing miRNAs exhibited an increased number of cells in the G1 phase (Fig. 7B and Supplementary Fig. S7A). (iii) The ability of a single adherent cell to survive and form colonies over a period of time was assayed through an in vitro colony formation assay. As displayed in Fig. 7C and Supplementary Fig. S7B, the colony formation ability was significantly reduced over two weeks in NB cells upon receiving miRs. (iv) Cell migration ability and neurosphere formation. A mechanical scratch was created on a monolayer of cultured cells at 0 h, and migration was measured in the number of pixels across the width of the wound after 20–36 h, depending on the growth of cells. As shown in Fig. 7D and Supplementary Fig. S7C, the closure of the wound over time was reduced in NB cells transfected with miRs. Additionally, Supplementary Fig. S7D demonstrates that overexpression of miRs resulted in the formation of small-sized neurospheres in all NB cells. Overall, miR-29a, miR-29b, and miR-29c inhibited NB cell growth, proliferation, colony formation, migration, and neurosphere formation.

Furthermore, using TargetScan and miRDB, we predicted miR-29a, miR-29b, and miR-29c interactions with CDKs, crucial in cell cycle phase transitions. CDK6 emerged as a target for all miR-29 family members, confirmed by our experiments showing CDK6 downregulation in NB cells with miR-29a, miR-29b, and miR-29c overexpression. We found diverse CDK regulation across cell lines: SK-N-BE(2)-C and SK-N-AS with miR-29 showed minimal changes, except for CDK2 downregulation in miR-29c-overexpressing SK-N-BE(2)-C; CHLA-255 displayed CDK4 and CDK6 downregulation, while SK-N-SH with miR-29b and miR-29c exhibited CDK4 and CDK2 downregulation, indicating unique regulatory profiles in NB cell lines (Supplementary Fig. S7E).

### MiR-29a, miR-29b, and miR-29c diminish the levels of tumor growth-promoting cytokines in NB tumors

**Fig. 8:**
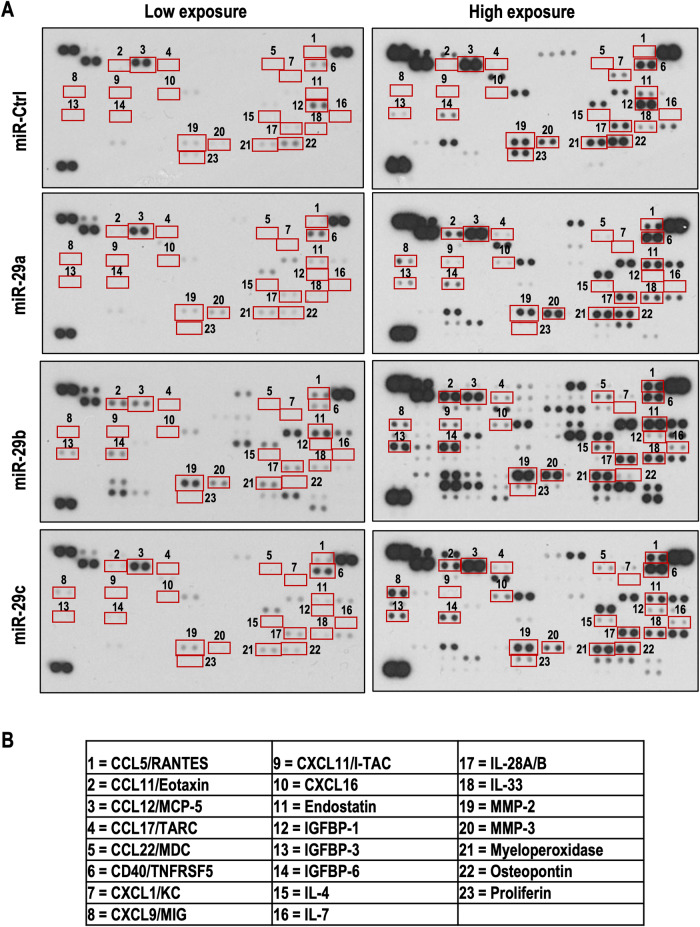
The expression profile of cytokines, chemokines, and growth factors. **A** The expression of cytokines, chemokines, and growth factors was analyzed in tumors from C57BL/6 mice that had received subcutaneous injections of 9464D cells stably expressing miR-Ctrl, miR-29a, miR-29b, and miR-29c. **B** The name of cytokines, chemokines, and growth factors were given.

Our analysis of cytokines, chemokines, and growth factors in tumors originating from mice injected with 9464D cells overexpressing miR-29a, miR-29b, and miR-29c is presented in Fig. 8 and Supplementary Fig. S8. In comparison to control tumors, those overexpressing miR-29 exhibited the following changes: (i) Elevated levels of anti-tumor cytokines such as CCL5 and CCL11, the transmembrane protein CD40, and the angiogenesis inhibitor endostatin. (ii) Upregulation of several chemokines (CXCL9, CXCL11, CXCL16) alongside downregulation of CXCL1. (iii) Variable regulation of insulin-like growth factor-binding proteins (IGFBPs), with IGFBP-1 downregulated and IGFBP-3 and IGFBP-6 upregulated. (iv) Increased expression of anti-tumor interleukins IL-4 and IL-7, as well as IL-28a and IL-33. (v) Elevated levels of matrix metalloproteinases (MMP-2 and MMP-3) and myeloperoxidase, a pivotal enzyme in the host-immune response. Additionally, downregulation of pro-tumorigenic osteopontin and proliferin was observed.

These results indicate that tumors overexpressing miR-29s exhibit heightened levels of multiple cytokines and chemokines associated with anti-tumor immunity.

## Discussion

The treatment of NB involves various conventional methods such as chemotherapy, surgical resection, autologous stem-cell transplant, radiation, and immunotherapy. Immunotherapy, specifically using the Dinutuximab antibody directed against GD2 (a disialoganglioside expressed on NB cells), has shown promise but comes with significant toxicity in high-risk NB patients [10, 11]. While CAR-T cell therapy has been explored in early phase clinical trials for NB, its efficacy is hindered by obstacles like insufficient T cell persistence and potency, a scarcity of tumor-specific targets, and an immunosuppressive tumor microenvironment [36–41]. Despite the success of immune checkpoint inhibitors in treating highly immunogenic adult solid tumors, they have not proven effective for NB patients due to factors such as low tumor mutation burden, limited MHC-I expression, infiltration by suppressive myeloid cells, and the production of inhibitory factors like arginase-2 and TGFβ [42–49]. Consequently, immune evasion poses a substantial challenge in effectively treating NB. In light of this, our proposal aims to identify and target the factors that regulate the immune response in NB to improve treatment outcomes.

Our clinical findings, based on multiple datasets, have revealed a significant correlation between higher B7-H3 expression and unfavorable outcomes in NB patients, including tumor relapse, disease progression, and poor histology. Our bioinformatic co-expression analysis on NB patient datasets highlighted the presence of exhaustive immune signatures, including B7-H3 in high-risk patients. In preclinical studies, inhibiting B7-H3 has shown promising results in restoring T and NK cell-mediated antitumor immunity and suppressing tumor growth [12, 14]. As we delve into understanding the mechanisms underlying these observations, we turned our attention to miRNAs. MiRNAs are known to regulate various oncogenic and tumor suppressive pathways by interacting with the 3′ UTRs of their target mRNAs, leading to post-transcriptional degradation or inhibition of protein translation [50]. However, the role of miRNAs in regulating the NK cell immune response and their association with patient survival in the context of NB has been limited. To address this gap, we investigated to determine whether miRNAs are involved in poor patient outcomes. Analyzing miRNA profiles in deceased NB patients from the TARGET patient dataset, we discovered that the miR-29 family members were notably depleted. This trend was particularly evident in stage 4 patients compared to those with stages 1–3. Higher expression levels of these miRNAs were positively correlated with better overall and event-free survival in NB patients. Furthermore, a decrease in miR-29a expression is strongly correlated with the upregulation of NK exhaustion genes, as observed in NB patient datasets. These findings provide a compelling rationale for further studying the impact of these miRNAs on immune evasion and poor patient survival in NB.

To explore the regulatory role of miRNAs on B7-H3 expression, we identified complementary binding sequences for miR-29a, miR-29b, and miR-29c on the B7H3 mRNA-3′UTR and an inverse association with B7H3 expression in patients, suggesting a potential mechanism of action. However, our investigation using TargetScan and miRDB miRNA databases did not reveal other immune checkpoint molecules PD-L1, CD155, or CD80/86 as targets of these miRNAs [25, 51–53]. Instead, both databases identified CD276 as a target of these miRNAs, with highly conserved binding sites. Additionally, when we assessed PD-L1 expression in the NB cell lines tested after miR-29s transfection, no significant difference was noted (Data not shown).

Understanding miR-29s and B7-H3 regulatory interactions may offer new insights into the molecular mechanisms that contribute to immune evasion and disease progression in NB. Therefore, we conducted experiments to explore the regulatory impact of miR-29a, miR-29b, and miR-29c on B7H3 expression. Through overexpression and inhibition experiments, we observed that these miRNAs effectively reduced both total and surface levels of B7H3 in various NB cell lines, including SK-N-BE(2)-C, SK-N-AS, CHLA-255, and SK-N-SH cells. Surface B7H3 on tumor cells has been shown to play a role in regulating an anti-tumor immune response by inhibiting the functions of immune cells, particularly NK cells and CD8+ T cells [14, 18, 54]. Therefore, we further investigated the effects of miR-29s on the anti-tumor immune response. It’s worth noting that NB tumors are predominantly characterized by low or negative expression of major histocompatibility complex (MHC), making it challenging for tumor-reactive CD8+ T cells to recognize and target these tumors, as they typically identify tumor antigens presented through the MHC I antigen presentation pathway [55]. On the other hand, NK cells do not strictly rely on MHC antigen presentation for their cytotoxic activity, enabling them to effectively target MHC-low tumors [56, 57]. As a result, NK cell-based immunotherapies are considered a promising approach to treating MHC-low tumors like NB. Given this context, we proceeded to examine the impact of miR-29s mediated B7H3 degradation on immune cell-mediated anti-tumor immune responses.

In our co-culture experiments, where we combined activated immune cells with NB cells expressing miR-29s, we observed higher expression levels of CD107a and perforin. This indicates the activation of NK cells upon co-culturing with NB cells expressing these miRNAs, compared to their respective control groups. CD107A, also known as lysosomal-associated membrane protein-1 (LAMP-1), serves as a marker for NK cell activation and protects NK cells from damage associated with degranulation [58]. Activated NK cells secrete increased levels of perforin, which creates pores in the target tumor cells’ membranes, facilitating NK cell cytolytic functions [59]. Additionally, our cytotoxicity assays revealed that NK cells displayed higher efficiency in lysing NB cells treated with miR-29s mimics. This suggests that the presence of miR-29s in NB cells enhances the sensitivity of these tumor cells to NK cell-mediated destruction.

Furthermore, we assessed the production of inflammatory cytokines IL-2 and TNFα in the culture medium. IL-2 secretion from activated NK cells plays a crucial role in antitumor activity as it recruits other immune cells to inflammatory sites and enhances the proliferation and cytotoxicity of NK cells during co-culture with tumor cells [60–62]. Interestingly, we found significantly higher secretion of IL-2 from NK cells co-cultured with miR-29a, miR-29b, and miR-29c expressing NB cells compared to miR-Ctrl cells [61–63]. This suggests that miR-29s may enhance the immune response against NB tumors by promoting the secretion of IL-2 and facilitating the recruitment and activation of other immune cells. On the other hand, TNFα, another cytokine, can have a dual role in the tumor microenvironment. While it can stimulate cancer cell growth, proliferation, invasion, metastasis, and tumor angiogenesis, it can also act as a killer of cancer cells [64]. The reduction in TNFα secretion observed in our experiments upon miR-29 expression further indicates that miR-29a, miR-29b, and miR-29c may inhibit tumor growth, at least in part, by downregulating TNFα.

Mechanistic experiments were conducted to understand the role of B7-H3 in the activation of NK cells and NK cell-mediated cytotoxicity, mediated by miR-29a, miR-29b, and miR-29c. These experiments involved interfering with B7-H3 through two approaches: blocking its surface using an anti-B7-H3 antibody or overexpressing B7-H3. In vitro assays evaluating the functions of effector NK cells, including cytotoxicity and cell invasion, revealed that NK cells displayed improved effectiveness in lysing and inhibiting the invasive capabilities of NB cells treated with miR-29 mimics. This enhanced efficacy can be attributed to the downregulation of B7-H3 expression by miR-29a, miR-29b, and miR-29c in NB cells and tumors. These findings suggest that treating NB cells with miR-29a, miR-29b, and miR-29c has the potential to enhance NK cell functionality and increase NK cell-mediated tumor lysis, particularly in tumors that overexpress B7-H3. Based on these promising in vitro results, the study aims to move forward and assess the effects of miR-29s in an in vivo setting to further validate their potential as therapeutic candidates.

In our study, we utilized murine NB cell lines 9464D and NB975, which were engineered to stably express miR-29s. These cells were then injected subcutaneously into an immune-competent C57/BL6 mouse model. The NB975 cell line expresses low levels of MHC-1, while 9464D has been previously shown to have minimal T cell infiltration [65]. The tumors derived from mice that received miR-29s expressing cells exhibited higher levels of perforin and the cytotoxic protease granzyme B in both NK cells and CD8+ T cells. This indicated that the tumors with miR-29s expression had a higher infiltration of activated immune cells compared to tumors expressing miR-Ctrls (control miRNAs). The increased perforin and granzyme B secretions are associated with the lysing of tumor cells by these activated immune cells, which was further evidenced by a marked reduction in tumor burden. Moreover, our data revealed that miR-29a, miR-29b, and miR-29c expressing tumors exhibited significant reductions in tumor vasculature, macrophage infiltration, and Ki67 (a marker of proliferation). In contrast, these tumors showed high levels of cleaved caspase-3, indicating increased apoptotic cell death. Macrophages are abundant immune cell populations in the tumor microenvironment and are known to support tumor growth by facilitating angiogenesis and immunosuppression [66–68]. Therefore, the inhibition of tumor vasculature and depletion of macrophages may represent additional anti-tumor mechanisms, making NB cells more sensitive to miR-29a, miR-29b, and miR-29c treatment. Additionally, our in vitro experiments further supported the notion that miR-29a, miR-29b, and miR-29c exert growth-suppressing effects. These miRNAs were found to inhibit cell proliferation, migration, invasion, and neural stemness. Further mechanistic insights indicate distinct regulation patterns of CDKs, including CDK6, CDK4, and CDK2, by miR-29 family members. These patterns may be attributed to the decrease in proliferation and other phenotypic changes observed after miR-29 treatment.

Moreover, in miR-29 overexpressing tumors, there were significant changes in cytokine and chemokine expression, such as upregulation of CCL5, CCL11, CXCL9, CXCL11, and CXCL16, known for their anti-tumor effects. These molecules inhibit tumor growth directly and enhance immune cell cytotoxicity against tumors [69–73]. Elevated levels of these cytokines and chemokines have been linked to the inhibition of B7-H3 and NK cell activation through anti-B7-H3 antibodies [72, 74–77]. Furthermore, antitumor CD40, IGFBPs like IGFBP-3 and IGFBP-6 and anti-angiogenic endostatin were upregulated in miR-29 overexpressing tumors. CD40 increase halts tumor growth via immune responses (T or NK-cell mediated), apoptosis, and antibody-dependent cellular cytotoxicity (ADCC) [78]. Combining CD40 agonist antibodies with immune checkpoint inhibitors enhances anti-tumor effects, suggesting miR-29 may improve immune-based NB therapies by downregulating B7-H3 and activating CD40 [78].

Additionally, the increased levels of anti-tumor interleukins IL-4, IL-7, IL-28a, and IL-33 in miR-29 overexpressing tumors suggest a role for these microRNAs in bolstering anti-tumor immunity. Combining these interleukins with CAR-T cell therapies has significantly improved T and NK-cell mediated antitumor immunity against tumors. [79, 80]. These findings hint at the possibility that the increased expression of these interleukins in miR-29-expressing tumors could contribute to heightened NK cell infiltration and activation within these tumor sites. Furthermore, the upregulation of MMP-2, MMP-3, and myeloperoxidase highlights the role of these tumors in host-immune interactions, while the decrease in osteopontin and proliferin suggests a shift toward a less favorable tumor microenvironment. This suggests increasing miR-29 expression could create a less favorable environment for tumor development.

A key limitation of our study is the use of subcutaneous NB tumor models, which may not fully mirror the tumor microenvironment around the adrenal glands, the typical origin site of NB. While our findings provide insights into miR-29’s effects on tumor immunity, future studies with orthotopic or patient-derived xenograft models could offer more accurate perspectives for understanding miR-29’s therapeutic potential in NB.

Collectively, our findings demonstrate that the expression of miR-29s in NB cells leads to increased infiltration of activated immune cells, enhanced cytotoxicity, reduced tumor vasculature, and macrophage infiltration, and increased apoptotic cell death, ultimately resulting in a marked reduction in tumor burden. These promising results provide a strong rationale for exploring the therapeutic potential of miR-29a, miR-29b, and miR-29c in NB treatment.

## Materials and methods

We used the following materials and methods in our study including: (1) Patient and PDX datasets, (2) Patient and PDX tumors, (3) Cell lines and culture conditions, (4) NK cell isolation, expansion, and culture, (5) miRNA transfections, (6) Lentiviral transductions, (7) Western blotting, (8) RNA Isolation and RT-qPCR, (9) Flow Cytometry, (10) Enzyme-Linked Immunosorbent Assay (ELISA), (11) The NK cell-mediated cytotoxicity assay by luminescence, (12) Fluorescence microscopy, (13) Immunohistochemistry (IHC) Staining, (14) Cell counting by trypan blue exclusion method, (15) Colony formation assay, (16) Wound healing assay, (17) Neurosphere-formation Assay, (18) Cytokine arrays, (19) NB mouse xenografts, and (20) Statistical analysis. The detailed methodology can be found in the supplementary materials and methods section.

### Supplementary information

Supplementary Figures and Methods

Uncropped images

## Data Availability

The data is available upon request.
