# Supplementary material for: The miR-29 family facilitates the activation of NK-cell immune responses by targeting the B7-H3 immune checkpoint in neuroblastoma
Source: Cell Death Dis. 2024 Jun 18;15(6):428. doi: 10.1038/s41419-024-06791-7 (PMC11189583; doi:10.1038/s41419-024-06791-7)
Supplement: Supplementary file 1 — Supplementary Figures and Methods [file 41419_2024_6791_MOESM1_ESM.pdf]

**Supplementary information**

# The miR-29 family facilitates the activation of NK-cell immune responses by targeting the B7-H3 immune checkpoint in neuroblastoma

Anup S. Pathania, Haritha Chava, Nagendra K. Chaturvedi, Srinivas Chava, Siddappa N. Byrareddy, Don W.Coulter, Kishore B. Challagundla

# Supplementary Figure S1

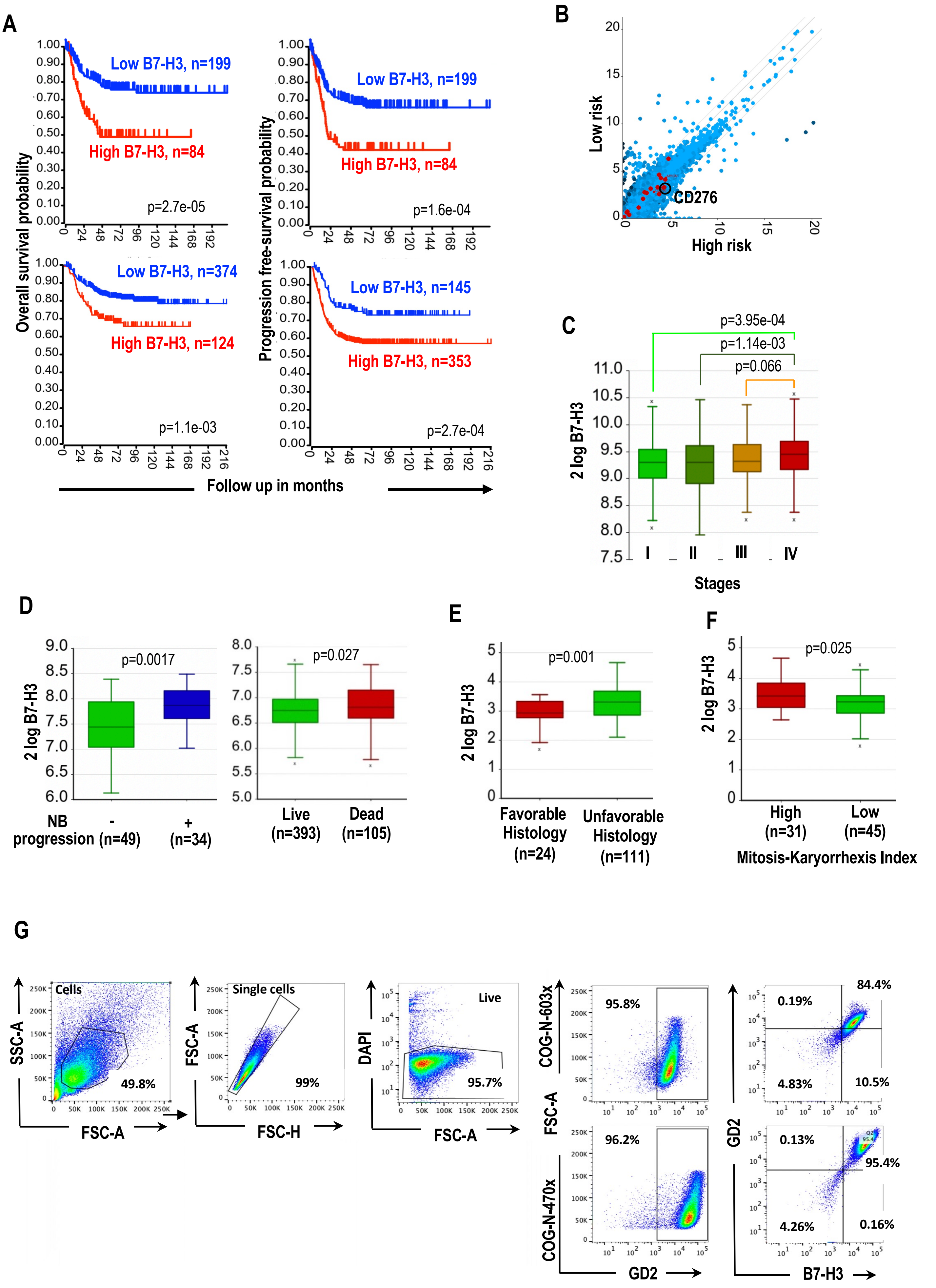

**Supplementary Fig. S1** (A) Kaplan-Meier curves demonstrate the correlation between different levels of B7-H3 expression and overall survival (left) and progression-free survival (right) in NB patients derived from the GSE85047 (n=283) (upper) and GSE62564 (n=498)(lower) datasets. Patients with higher B7-H3 expression had shorter survival. (B) An X-Y plot compares the gene expression of B7-H3 in high-risk and low-risk NB patients (n=498) using data from the GSE62564 RNA-seq dataset. The circled dot on the plot indicates the distribution of B7-H3 expression, which is shifted toward the Y-axis, suggesting elevated expression in the high-risk group compared to the low-risk group. Other dots represent the expression of genes involved in the negative regulation of neural precursor cell proliferation. (C-F) Box plots represent the gene expression of B7-H3 in stage 4 vs. stages 1-3 (GSE45547, n=649) (C), higher vs. lower progression (GSE16476, n=88) (D), deceased vs. alive (GSE62564, n=498) (D), unfavorable vs. favorable histology groups and higher vs. lower Mitosis-Karyorrhexis Index (MKI) (TARGET) (E, F) in NB patients from different datasets. Patients with stage 4, higher progression, deceased, unfavorable histology, and higher MKI significantly exhibited higher expression of B7-H3 compared to their respective controls. (G) Representative flow cytometric plots show the surface expression of B7-H3 on GD2-positive NB tumor cells isolated from PDX tumor tissues of patients at the diagnosis and relapse stages. The plots display the percentage of B7-H3-positive cells in each quadrant. The gating strategy of the GD2-enriched PDX-derived tumor cell population was achieved through cell sorting by flow cytometry using PE-GD2 antibodies.

# Supplementary Figure S2

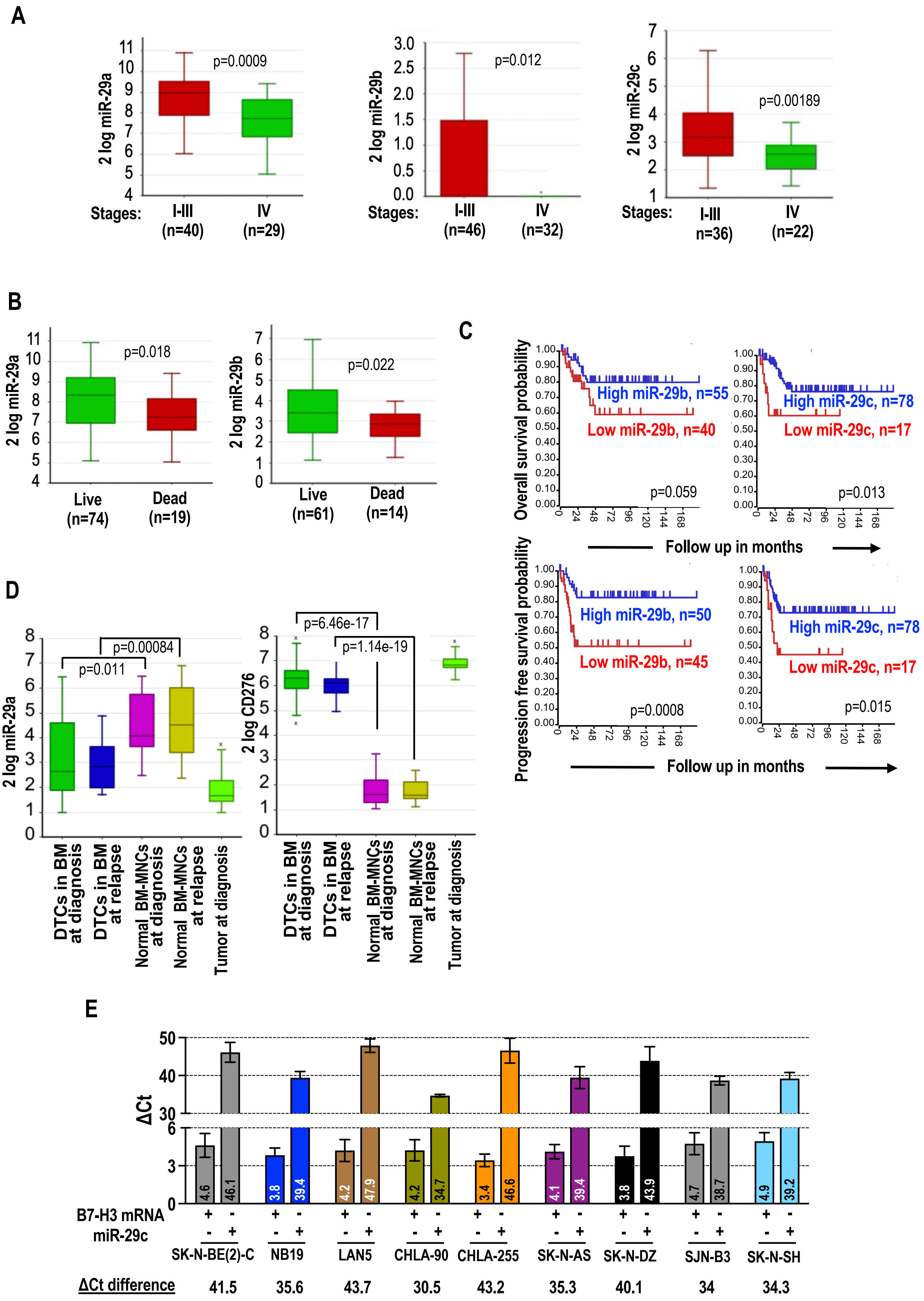

**Supplementary Fig. S2** (A) Box plots depict the expression levels of miR-29a, miR-29b, and miR-29c in stage 4 NB patients compared to stages 1-3 NB patients from the GSE155945 (n=97) dataset. (B) Box plots illustrate the expression levels of miR-29a and miR-29b in deceased NB patients compared to those who survived from the GSE155945 (n=97). (C) Kaplan-Meier curves display the association between varying levels of miR-29b and miR-29c and overall survival (left) and progression-free survival (right) in NB patients using data from the Tumor GSE155945 (n=97) dataset. Patients with elevated miR-29 expression showed improved survival outcomes. (D) The correlation between the expression levels of miR-29a (on the left) and B7-H3 (on the right) in disseminated tumor cells (DTCs) and normal bone marrow-derived mononuclear cells (MNCs) among stage 4 metastatic NB patients derived from the GSE94035 (n=86) dataset. Expression levels of miR-29a and B7-H3 in the primary NB tumors at the time of diagnosis were also provided. (E) The graphs illustrate the physiological expression levels of B7-H3 mRNA and miR-29c in nine distinct NB cell lines. These results are presented as the mean ± standard error (SEM) and are derived from 3-4 independent biological experiments.

# Supplementary Figure S3

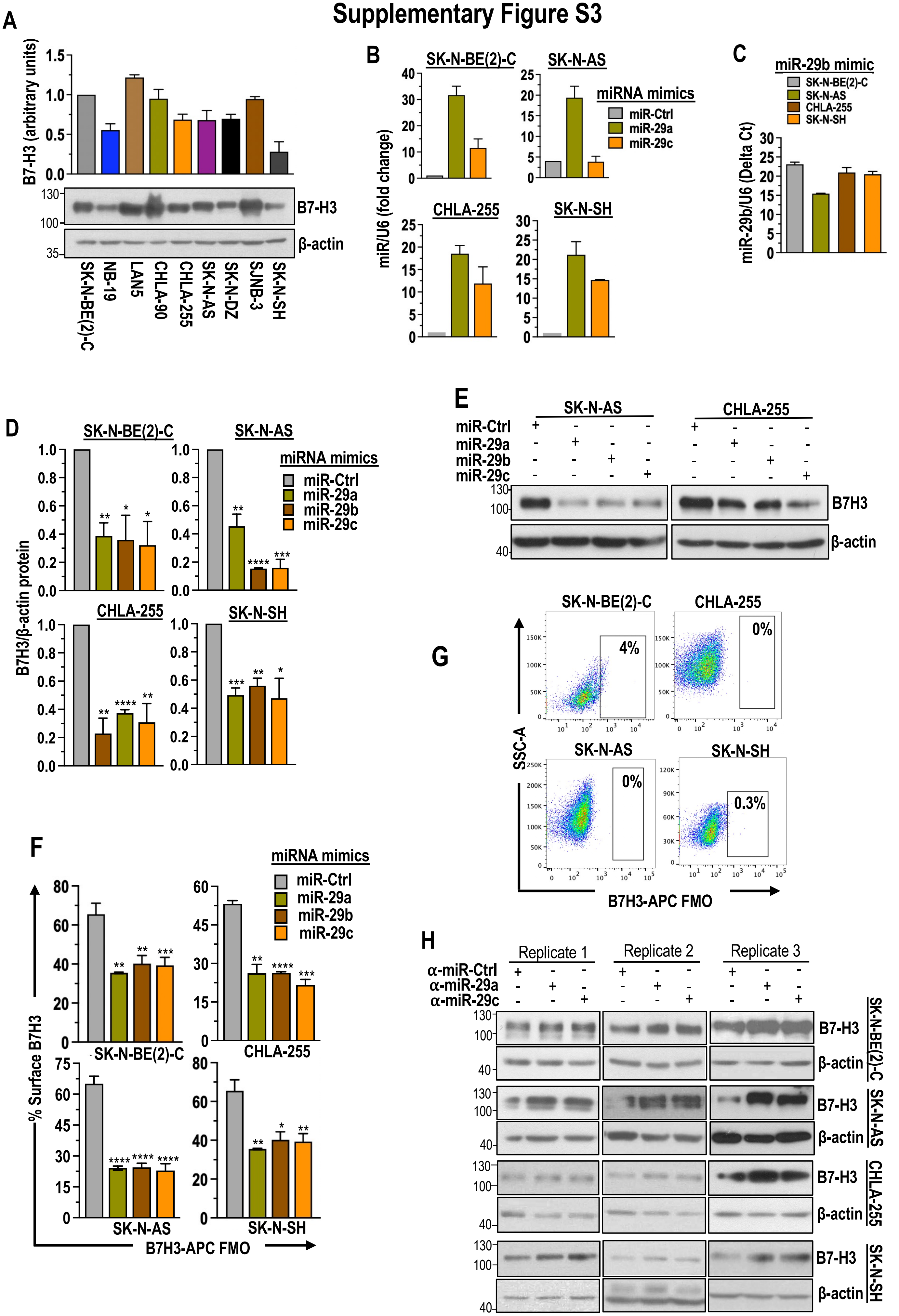

**Supplementary Fig. S3** (A) Western blot analysis of B7-H3 protein levels in whole lysates of nine different NB cell lines. B7-H3 levels were quantified using densitometric analysis, normalized to β-actin, and presented as arbitrary units. (B) A qRT-PCR expression presented as the fold change of miR-29a or miR-29c, and (C) delta Ct values of miR-29b normalized to U6 snRNA in NB cell lines. (D) Densitometric quantification graph depicting the relative B7-H3 protein levels normalized to the control group. (E) Western blot analysis of B7-H3 total protein levels in stable NB cells expressing miR-29a, miR-29b, and miR-29c, or miR ctrl mimics for 48 h. (F) Representative quantification graphs showing surface B7-H3 protein levels in NB cells transfected with miR-29a, miR-29b, and miR-29c, or miR control mimics for 48 hours. (G) Representative flow cytometric plots demonstrating fluorescence-minus-one (FMO) controls of NB cells stained with all fluorochromes except for B7-H3, used to set the background signal for figure 3B. (H) Replicates of Western blotting for B7-H3 total protein in NB cell lines shown in Figure 3D, treated with inhibitors of miRs such as $a$-miR-29a, $a$-miR-29c, or $a$-ctrl miRs for 48 hours. β-actin was used as an internal loading control to normalize B7-H3 expression. Data are presented as mean ± standard error from three to four independent biological replicates. Statistical analysis was performed using a two-sided unpaired t-test. *$p < 0.05$, **$p < 0.01$, ***$p < 0.001$, and ****$p < 0.0001$.

# Supplementary Figure S4

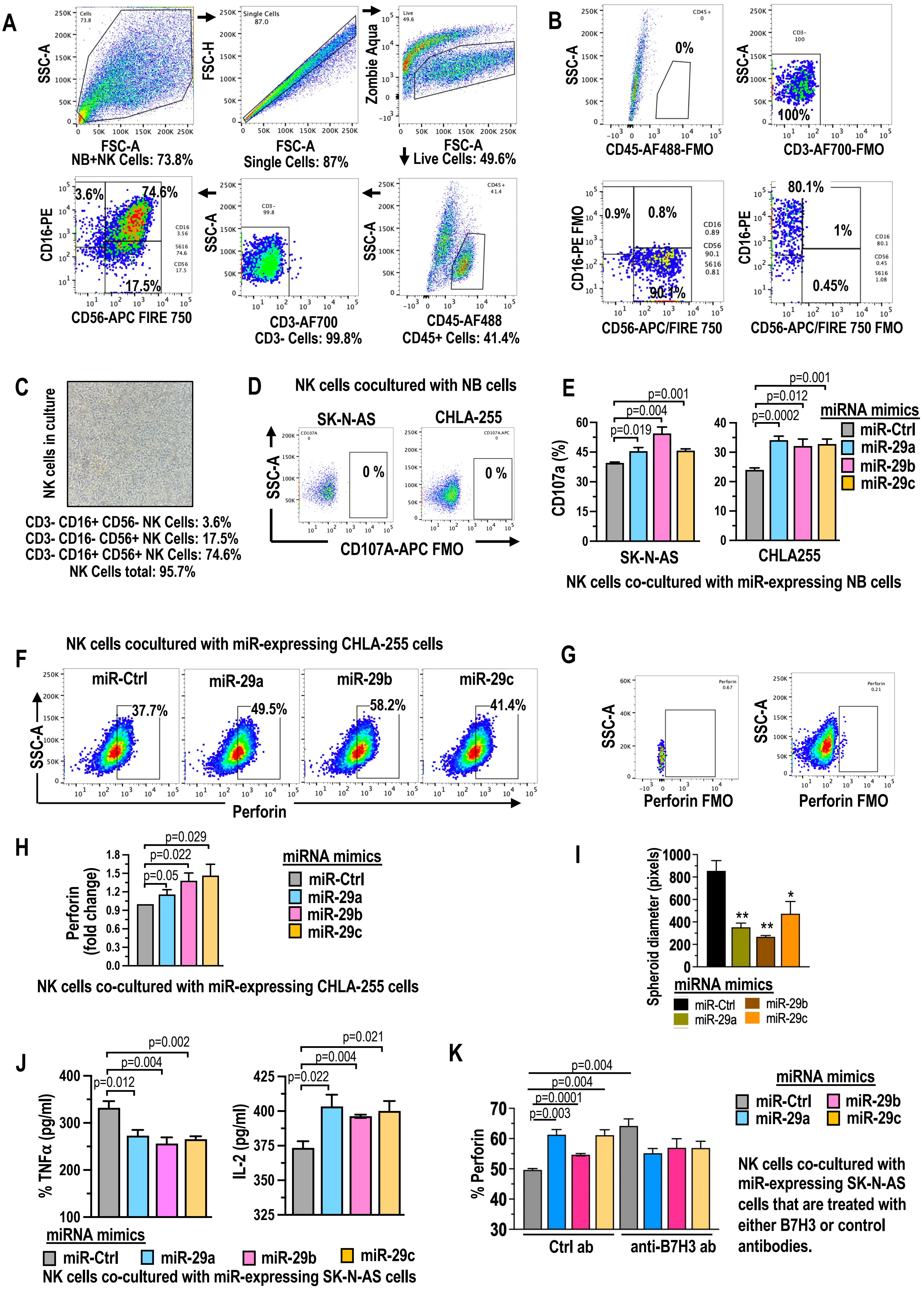

**Supplementary Fig. S4 (A, B)** Representative flow cytometry plots illustrate the gating strategy employed for the isolation of NK cells (A) and the used FMO controls (B). NK cells were stained with CD3-AF700, CD56-APC/Fire750, and CD16-PE antibodies, and CD3-CD56+CD16+ NK cells were isolated. (C) The phase-contrast image corresponds to NK cells in culture. (D) Representative flow cytometry plots display the FMO controls for CD107A-APC staining in NK cells cocultured with NB cells, related to Figure 4A. (E) The quantification graphs display the percentage of CD107+ NK cells that are cocultured with miR-expressing NB cells, related to Figure 4A. The data were derived from four similar experiments. Bar graphs represent the mean and SEM values. (F-H) Representative flow cytometry plots illustrate the expression of perforin in NK cells co-cultured (E:T=1:2 for 5 h) with CHLA-255 cells that were transfected with miR-29a, miR-29b, miR-29c, or miR Ctrl mimics for 48 h (F) and the used FMO controls of perforin-PE/DAZZLE (G). The quantification graphs display the percentage of perforin+ NK cells. Bar graphs represent the mean and SEM values (H). (I) Quantification graphs of spheroid diameters in SK-N-B(E)2 spheroids stably expressing miR-29a, miR-29b, and miR-29c, co-cultured (E:T=2:1) with IL-15-treated NK cells for 48 h. Spheroid diameters were measured in pixels using Photoshop. P-values were calculated using a two-tailed unpaired Student's t-test. Bar graphs represent the mean±SEM of 3 biological replicates. (J) ELISA assay-based quantification graphs of TNF-α and IL-2 measured from the culture medium of SK-N-AS cells, which were transfected with miR-29a, miR-29b, miR-29c, or miR-Ctrl mimics for 48 hours and co-cultured with activated NK cells for 5 hours (E:T=1:2). (K) The quantification graphs display the percentage of perforin+ NK cells co-cultured (E:T=1:2 for 5 h) with NB cells pretreated with an anti-B7-H3 antibody (5 μg) for 24 h and transfected with miR-29a, miR-29b, miR-29c, or miR Ctrl mimics for an additional 24 h. The data were derived from four similar experiments. Bar graphs represent the mean and SEM values.

# Supplementary Figure S5

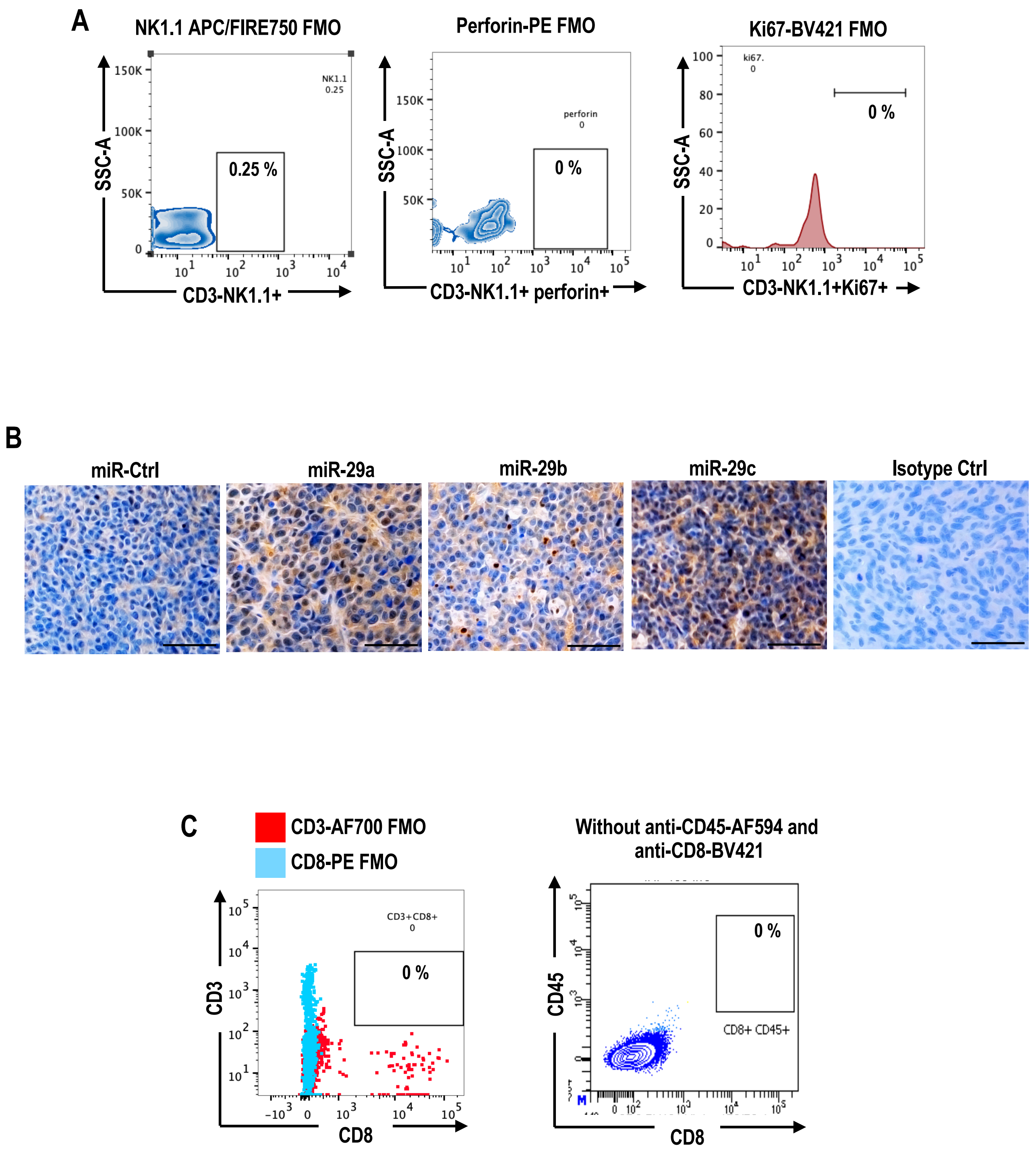

**Supplementary Fig. S5** (A) Representative flow cytometry plot showing the fluorescence-minus-one (FMO) control for NK1.1 APC/FIRE750, perforin-PE and Ki67-BV421. These FMO controls were utilized to establish proper gating strategies and ensure accurate interpretation of the flow cytometry data presented in Figure 5D. (B) IHC images displaying CD161c/NK1.1 expression. These images provide visual evidence of NK cell infiltration in tumor tissues obtained from C57BL/6 mice that received a single injection of murine 9464D cells expressing GFP-miR-29a, miR-29b, miR-29c, or GFP-control (ctrl) miRs and were observed for 30 days. (C) CD3-AF700, CD8-PE, CD45-AF594 and CD8-BV421FMO controls used for gating strategies in Figure 5F.

# Supplementary Figure S6

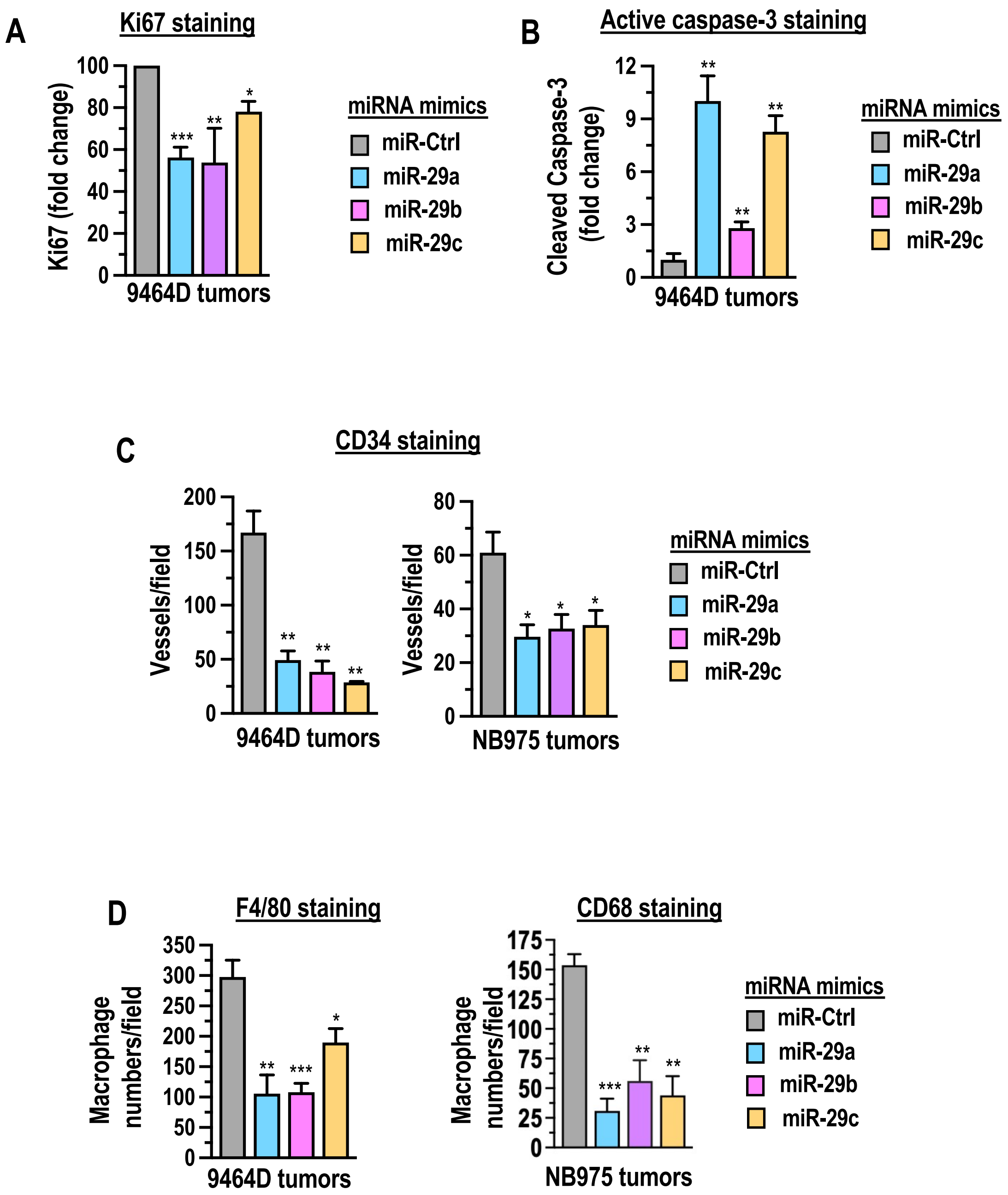

**Supplementary Fig. S6** The quantification graphs show the- (A) fold change in Ki67 expression, a marker of cellular proliferation, (B) fold change in cleaved caspase-3 expression, a marker of apoptosis, (C) average number of vessels observed per field, (D) number of macrophages observed per field in tumors derived from mice subcutaneously injected with 9464D and NB975 cells stably expressing miR-29a, miR-29b, miR-29c, or miR-Ctrl. The analyses of the quantifications were carried out in tumors (n=4) using the digital imaging count tool in Adobe Photoshop. Bar graphs represent the mean ± standard error of the mean (SEM).

# Supplementary Figure S7

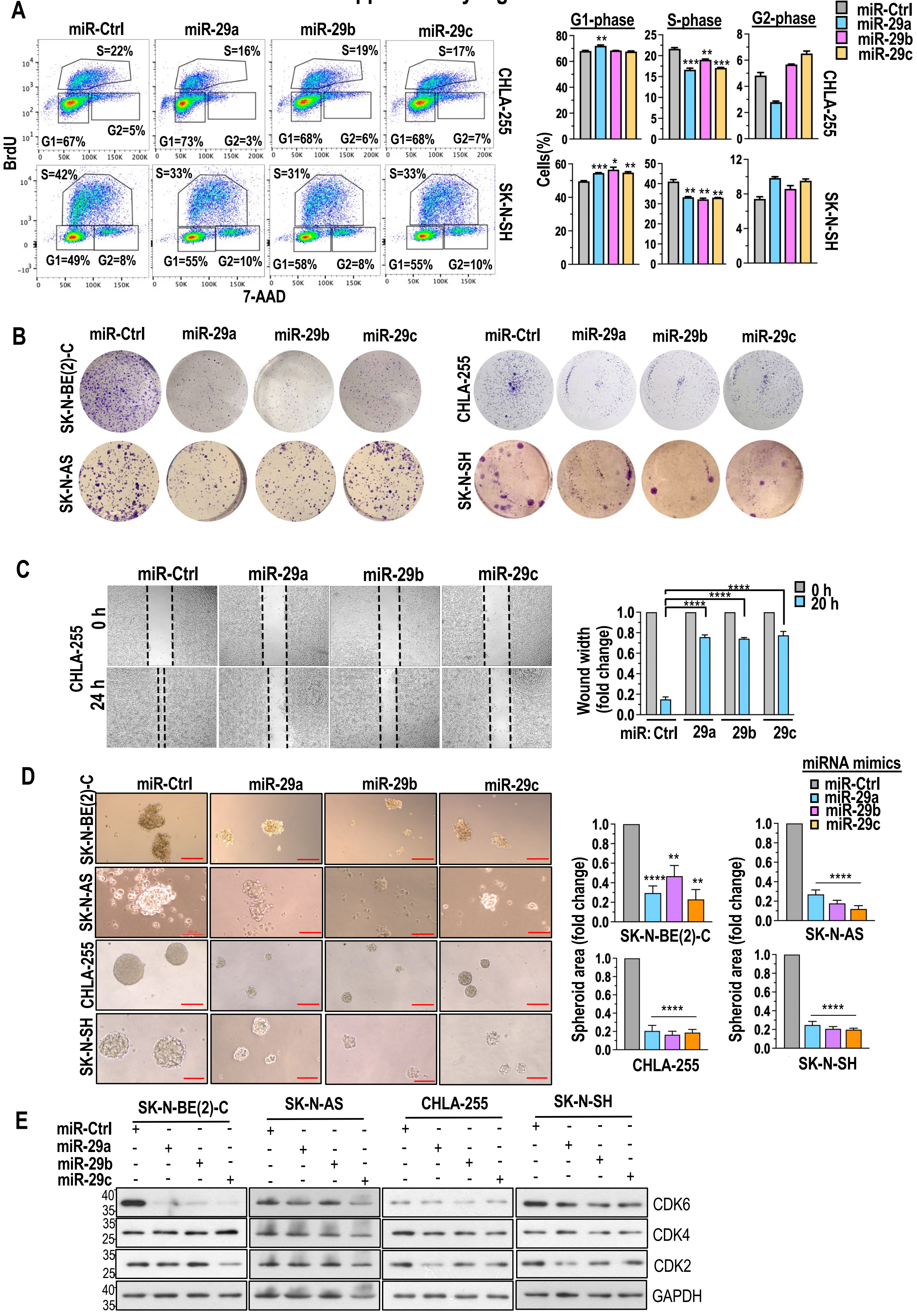

**Supplementary Fig. S7** NB cells were transfected with miR-29a, miR-29b, miR-29c, or a miR-Ctrl for a duration of 48 hours. (A) BrdU incorporation assay, combined with DNA dye 7-AAD, was used for cell cycle analysis. Flow cytometry plots depict the percentage of BrdU-positive cells, representing those in S-phase, as well as G1 and G2 phases of the cell cycle, analyzed through 7-AAD staining, accompanied by a graph quantifying the percentage of cells in each phase. (B) Images of colony formation illustrating the formation of colonies, along with accompanying quantification graphs showing the number of colonies per well. (C) Representative images of wound healing assays and a quantification graph demonstrating wound width in CHLA-255 cells (D) Representative micrographs of neurospheres from NB cells stably expressing miR-Ctrl, miR-29a, miR-29b, or miR-29c. The spheroid area was measured in pixels using digital imaging (Photoshop), and fold change differences between miR-Ctrl and miR-29-expressing NB cells were calculated. P-values were calculated using a two-tailed unpaired Student's t-test. Bar graphs represent mean ± SEM (n=3 biological replicates). (E) Western blotting images displaying the levels of cell cycle proteins in NB cells transfected with miR-29a, miR-29b, and miR-29c, or miR Ctrl mimics for 48 hours. GAPDH was used as the internal control.

## Supplementary Figure S8

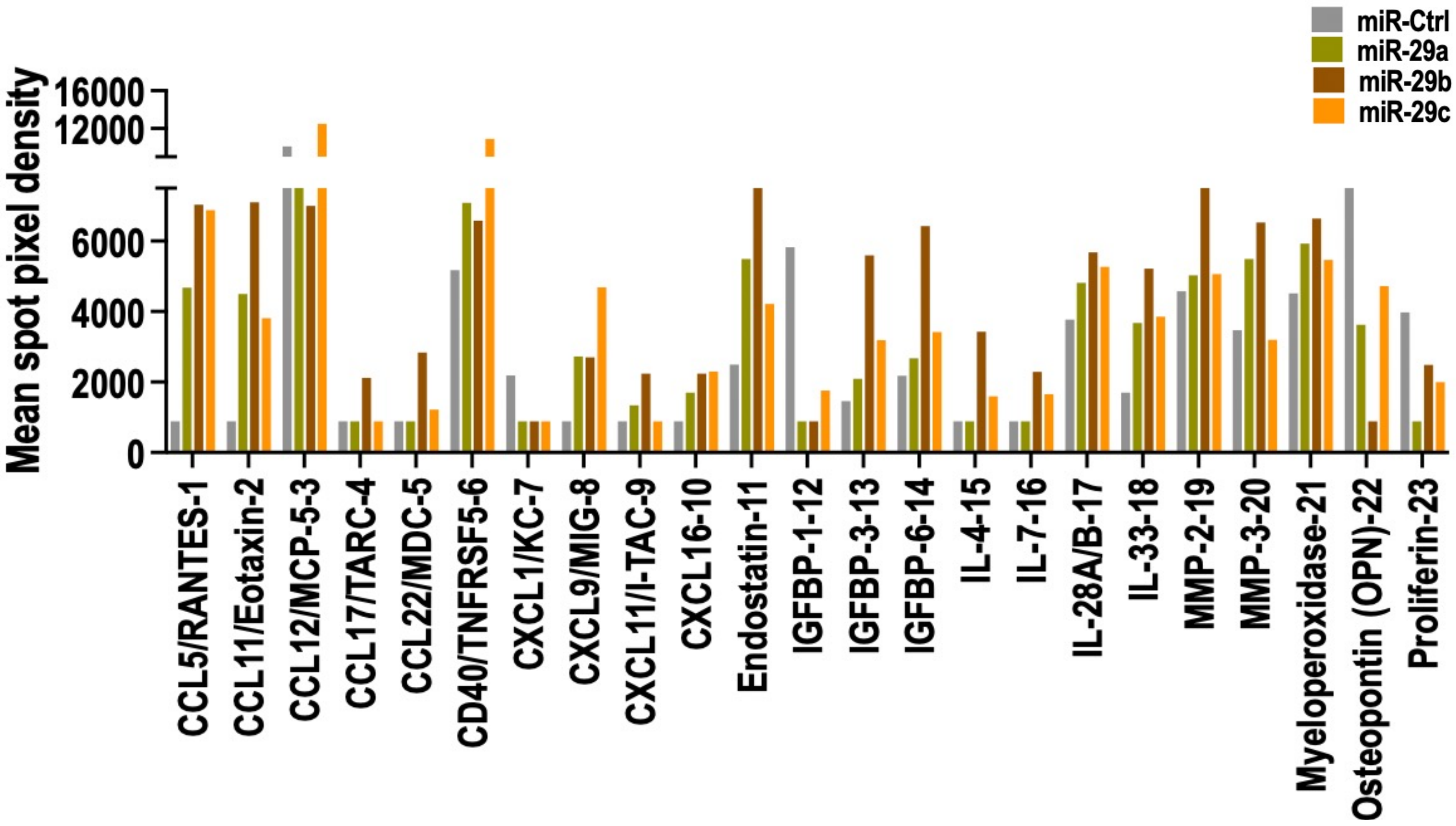

**Supplementary Fig. S8** The graph showing the quantification of cytokines, chemokines, and growth factors analyzed in tumors from C57BL/6 mice that had received subcutaneous injections of 9464D cells stably expressing miR-Ctrl, miR-29a, miR-29b, and miR-29c. The bar graphs depict the mean spot pixel density. The data were obtained using a transmission-mode scanner and subsequently analyzed using Image Studio image analysis software.

## SUPPLEMENTARY MATERIALS AND METHODS

**Patient and PDX datasets**. An overview of the datasets and analysis methods used in the study is given below. (1) NB TARGET dataset: The NB TARGET dataset, consisting of 151 samples, was downloaded from the National Cancer Institute's website (https://ocg.cancer.gov/programs/target/projects/neuroblastoma). (2) B7-H3 dependent Kaplan-Meier survival analysis: Three datasets with GSE numbers GSE16476 (n=88)[1], GSE85047 (n=283) [2], and GSE62564 (n=498) were used. These datasets contained relevant information for assessing the impact of B7-H3 on patient survival. (3) miR-29a, miR-29b, and miR-29c based Kaplan-Meier survival analysis: The Tumor NB - Bell - 97 - tmm - mirbase22 (GSE155945, n=97) dataset was used for Kaplan-Meier survival analysis related to miR-29a, miR-29b, and miR-29c [3]. This dataset provided the necessary data to examine the survival outcomes associated with these specific miRNAs. (4) Gene expression analysis: For B7-H3 gene expression analysis, the datasets TARGET (n=151), GSE62564 (n=498), GSE45547 (n=649)[4], and GSE16476 (n=88)[1] were utilized. (5) miRNAs expression analysis: For changes in miRNAs analysis, the TARGET (n=151) dataset was utilized. For miR-29a, miR-29b, and miR-29c expression analysis, the datasets: Tumor NB - Bell - 97 - tmm - mirbase22 (GSE155945, n=97)[3], GSE94035 (n=86)[5] were used. For B7H3 and miR29 correlation the dataset, Exp NB (TAE-684 / shALK) - de Preter - 113 - RMA - u133p2 was utilized[6]. (6) Analysis platform: The data sets mentioned above were analyzed using R programming and R2, a web-based genomics analysis and visualization application platform (http://r2.amc.nl).

**Patient and PDX tumors.** The patient and PDX tumors were utilized in the study as described below: Patient (1) Tissue Microarray (TMA): Deidentified formalin-fixed, paraffin-embedded (FFPE) whole tissue sections with a thickness of 5 µm were obtained as TMA slides (cat# NB642a) from US Biomax Inc, now TissueArray.Com LLC. Each TMA slide contained 26 patient samples and 5 normal peripheral nerve tissues, all in duplicates. (2) PDX Tumors: We have established NB-specific PDX tumors in collaboration with C. Patrick Reynolds, MD, PhD, from Texas Tech University Health Sciences Center through the Alex's Lemonade Stand Foundation (ALSF)/Children's Oncology Group (COG) Childhood Cancer Repository and the Childhood Solid Tumor Network at St. Jude Children's Research Hospital as we described earlier [7]. The deidentified human tumor materials were used in accordance with the Declaration of Helsinki. All methods were carried out in accordance with relevant guidelines and regulations approved by the UNMC. Briefly, NB-specific patient tumor samples were surgically removed and cut into multiple pieces in sterile Hanks' balanced salt solution supplemented with antibiotics. The deidentified patient tumor cells were subcutaneously injected into nude mice aged 4 to 6 weeks. The mice were observed for the development of palpable tumors, and the tumor tissues were harvested once the tumors reached the desired volume. A single-cell suspension was prepared from the harvested tumor tissues for reimplantation into the next set of mice, allowing for multiple passages and expansion of the PDX tumors. The established PDX tumor tissues, including SJNBL012407_x1, SJNBL013763_x1, COG-N-424x, COG-N-470x, COG-N-496x, COG-N-603x, COG-N-484, and COG-N-564x were used in the study. These patient tissue microarray blocks and PDX tumor tissues/blocks were employed for western blotting and immunohistochemical (IHC) analysis. The study methodologies involving patient and PDX tumor tissues were approved by the institutional ethics committee.

**Cell lines and culture conditions.** The following human and murine cell lines were used in the study. (1) Human NB cell lines: SK-N-BE(2)-C, SK-N-AS, SK-N-SH, CHLA-255, NB-19, LAN5, CHLA-90, SK-N-DZ, and SJNB-3. These cell lines were obtained from the American Type Culture Collection (ATCC) or the Children's Oncology Group Cell Culture and Xenograft Repository (www.cogcell.org). (2) Murine cell lines: NB975 and 9464D. NB975 was kindly provided by Dr. Leonid Metelitsa, MD, Ph.D., from Texas Children's Hospital in Houston, Texas. 9464D was generously provided by Dr. Paul M. Sondel, MD, Ph.D., from the University of Wisconsin-Madison in Madison, Wisconsin. (3) The CSTX002 (feeder K562.mbIL21.4-1BBL) cell line that genetically modified to express membrane-bound IL-21 (mbIL21) and 4-1BB Ligand (CD137L), was a generous gift from Dr. Siddappa Byrareddy, Ph.D., from UNMC. SK-N-BE(2)-C, NB-19, LAN5, SJNB-3, NB975, and CSTX002 cells were cultured in Roswell Park Memorial Institute (RPMI) medium. SK-N-AS, SK-N-DZ, and 9464D cells were cultured in Dulbecco's Modified Eagle Medium (DMEM) medium. SK-N-SH cells were cultured in Minimum Essential Medium (MEM). CHLA-255 and CHLA-90 cells were grown in Iscove's Modified Dulbecco's Medium (IMDM). All media were supplemented with 10% heat-inactivated fetal bovine serum (FBS; Sigma, cat# F0926), 2 mM glutamine, 50 U/mL penicillin/streptomycin (Gibco, cat# 15070063), and MEM non-essential amino acids (Gibco, cat# 11140050). All cell lines were cultured in a humidified $CO_2$ incubator with 5% $CO_2$ at 37°C. The cell lines were authenticated using short tandem repeats (STR) analysis, and they were tested to ensure they were free of mycoplasma contamination prior to conducting experiments.

**NK cell isolation, expansion, and culture.** Human peripheral blood mononuclear cells (PBMCs) were obtained from the institutional Elutriation Core Facility. The MojoSort Human NK Cell Isolation Kit (Biolegend, cat#480054) was used for NK cell isolation, following previously established protocols [7]. Fresh PBMCs ($1x10^7$) were suspended in 100 µL of MojoSort buffer (BioLegend, cat#480017) and incubated with 10 µL of the Biotin-Antibody Cocktail for 15 minutes on ice. The cells were washed with 4 ml of MojoSort buffer, centrifuged for 5 minutes at 300xg and then resuspended in 100 µL of MojoSort buffer. Streptavidin magnetic nanobeads (10 µL) were added to the cell suspension and incubated for 15 minutes on ice. After incubation, the cells were washed with 4 ml of MojoSort buffer, resuspended in 2.5 ml of MojoSort buffer, and placed in a magnet for 5 minutes at RT. This step allowed the untouched NK cells to be separated by pouring the liquid into a fresh tube. The isolated NK cells were cultured in RPMI medium supplemented with 10% fetal bovine serum (FBS), interleukin-2 (IL-2) at a concentration of 20 ng/ml, and IL-15 at a concentration of 50 ng/ml for a period of four days and used for the expansion. The obtained NK cells were co-cultured with irradiated K562 CSTX002 mbIL21.41bbL cells at a 1:1 ratio and supplemented with IL-2 at a concentration of 20 ng/ml. This co-culture was maintained for two weeks. The NK cells were replenished with fresh K562 cells every week and with recombinant human IL-2 (rhIL-2) twice a week to support their growth and expansion. The purity of the expanded NK cells was determined by flow cytometry using NK cell marker anti-CD56 and anti-CD16 antibodies. The presence of T-cell contamination was checked using an anti-CD3 antibody. The expanded NK cells were identified as CD56+CD16+CD3- cells, indicating the successful expansion of NK cells (Supplementary Figure 4A). These cells were then used for coculture experiments with NB cells or preserved by freezing in liquid nitrogen using a freezing medium containing 95% FBS and 5% dimethyl sulfoxide (DMSO) for future use.

**miRNA transfections.** The miRNA mimics, including hsa-miR-29a-3p (cat# 4464084, ID: MH12499), hsa-miR-29b-3p (cat# 4464066, ID: MC10103), hsa-miR-29c-3p (cat# 4464066, ID: MC10518), and the miRNA inhibitors, such as hsa-anti-miR-29a (cat# 4464084, ID: MH12499) and hsa-anti-miR-29c (cat# 4464084, ID: MH10518), as well as their corresponding negative controls, were obtained from Life Technologies. NB cells were initially cultured at a density of $0.09 \times 10^6$ cells in 3 mL of 10% FCS medium for 24 hours to allow them to adhere and settle. Next, the medium was replaced with Opti-MEM reduced serum medium (ThermoFisher Scientific, cat# 31985070) to minimize serum interference. The cells were then transfected with the miRNAs of interest at a concentration of 60 nM using lipofectamine RNAiMAX (ThermoFisher Scientific, cat# 13778150) as the transfection reagent. The transfection was performed for 8 hours to facilitate miRNA uptake by the cells. Following the transfection, the medium was replaced with fresh DMEM containing 10% FCS. The cells were then allowed to grow for an additional 24 to 48 hours.

**Lentiviral transductions.** Lentiviral vectors carrying human precursor (pre)-miR-29a (cat# PMIRH29aPA-2), human pre-miR-29b (cat# PMIRH29b-1PA-1), human pre-miR-29c (cat# PMIRH29cPA-1), nonfunctional control pre-miR (cat# PMIRH000PA-1), mouse pre-miR-29a (cat# PMIRH29cPA-1), mouse pre-miR-29b (cat# MMIR-29b-1-PA-1), mouse pre-miR-29c (cat# MMIR-29c-PA-1), and their nonfunctional pre-miR control (cat# MMIR-000-PA-1) were obtained from System Biosciences (Palo Alto, CA). To generate lentiviral particles, 293T cells were cultured at a density of $3 \times 10^6$ cells in 8 mL of 10% FCS DMEM medium for 24 hours. The medium was then replaced with serum-free medium, and the cells were co-transfected with lentiviral miRNA-expressing plasmids (10 µg) and packaging plasmids including pVSV-G (5 µg), pMDL (10 µg), and pREV (5 µg) using polyethyleneimine (PEI) transfection reagent. The DNA to PEI ratio used was 1:4 (µg/µL). After 24 hours of transfection, the medium was replaced with fresh DMEM containing 10% FCS, and the cells were allowed to grow for 48 to 72 hours. The lentiviral supernatants were collected and centrifuged at 1000 g for 5 minutes, followed by filtration through a 0.45-µm syringe filter to remove cellular debris. The lentiviral particles were concentrated using PEG-it virus precipitation solution (System Biosciences, cat# LV810A-1/LV825A-1) according to the manufacturer's protocol. The concentrated lentiviral particles were used for infecting NB cells. NB cells were plated at a density of $1 \times 10^5$ cells per well in a 6-well plate with 3 mL of growth medium, allowing them to reach 40% confluency over 24 hours. The cells were then infected with the lentivirus for 24 hours. After that, the medium was replaced with fresh regular growth medium, and the cells were allowed to grow for 3 to 5 days. To select the transduced cells, puromycin (5 µg/mL) was added to the growth medium, or GFP-positive cells were sorted using flow cytometry as we described earlier [7]. The selected and transduced cells were used for downstream experiments.

**Western blotting.** Cells were lysed using radioimmunoprecipitation assay (RIPA) buffer (ThermoFisher, cat#89901) with freshly added EDTA-free protease and phosphatase inhibitor tablets (ThermoFisher, cat#A32961) for 30 minutes on ice. The cell lysates were then centrifuged at 13,000 rpm for 30 minutes to obtain cleared supernatants. The protein concentration of the lysates was determined using the Bradford Protein Assay (Bio-Rad) and an equal amount of proteins were loaded onto 10% or 12% gradient SDS-PAGE gels. The proteins were resolved through electrophoresis and transferred onto a nitrocellulose membrane followed by blocked with 5% BSA for 1 hour at RT [8]. The membrane was incubated overnight at 4°C with primary antibodies: anti-B7-H3 monoclonal antibody (Proteintech, cat#66481) at a dilution of 1:6000 and

anti-GAPDH antibody (Proteintech, cat#60004-1-Ig) at a dilution of 1:250,000. After incubation with primary antibodies, the membrane was incubated with HRP (horseradish peroxidase)-conjugated secondary antibodies at RT for 1 hour and developed using an enhanced chemiluminescent reagent. The resulting protein bands were captured on X-ray film and quantification was performed using Image Studio Lite software (LI-COR Biosciences).

**RNA isolation and reverse transcription-quantitative real-time PCR (RT-qPCR).** Total RNA extraction from cells was performed using the Pure-Link RNA Mini Kit (Life Technologies, cat#12183025), following the manufacturer's instructions. The reverse transcription (RT) reactions were conducted using 500-1000 ng of total RNA using the iScript cDNA synthesis kit (Bio-Rad, cat#4366597) for mRNA analysis, whereas TaqMan microRNA Reverse Transcription kit (Applied Biosystems, cat#4440041) for miRNA analysis. The resulting cDNAs were subjected to amplification by RT-qPCR using either the ABI StepOne RT-qPCR system (Applied Biosystems) or the Bio-Rad CFX96 Real-Time PCR Detection system. For mRNA analysis, SYBR green mix (Bio-Rad) and for miRNA analysis, TaqMan Universal master mix II (Life Technologies, cat#4440041) were used as detection reagents. To normalize the expression levels, relative mRNA expression was normalized to GAPDH mRNA, while miRNA expression was normalized to U6 small nucleolar RNA (U6 snRNA). Changes in fold amplification were determined using the ΔΔCt method, which calculates the difference in threshold cycle values between the target gene and the reference gene [9]. The qRT-PCR primer sequences for B7-H3 and GAPDH were purchased from Integrated DNA Technologies, Inc. The sequences are as follows: B7-H3 Forward: GCACGGTTACACAGAGGGAC, Reverse: GCCCAGGAGTGGATTGATCAGCC. GAPDH Forward: 5'-GATTCCACCCATGGCAAATTC-3', Reverse: 5'-AGCATCGCCCCACTTGATT-3'. The qRT-PCR primer sequences for miR-29a, miR-29b, miR-29c, and U6 snRNA were purchased from Life Technologies (cat#4427975, ID: 002112, ID: 000413, ID: 000587, and ID: 001973, respectively).

**Flow cytometry.** Cells ($1x10^5$) were collected by centrifugation at 350g for 5 minutes, washed with PBS, and then resuspended in PBS at a concentration of $1.0x10^5$ cells per 100 µL. To exclude dead cells, the cell suspension was incubated with Zombie Aqua fixable viability dye (BioLegend, #423102) at RT for 30 minutes. The cells were washed twice with cell staining buffer (BD Biosciences, cat#554656). Fc receptors were blocked by incubating the cells with Human TruStain FcX antibody (BioLegend, cat#422302) for human cells or TruStain FcX PLUS (anti-mouse CD16/32) antibody (BioLegend, cat#156604) for mouse cells for 15 minutes on ice, reducing non-specific antibody binding. Fluorochrome-conjugated antibodies targeting cell surface proteins were added to the cell suspension and incubated for 20 minutes on ice to stain the cells. After incubation, the cells were washed twice with staining buffer, fixed, and permeabilized using cyto-Fast Fix/Perm Buffer (BioLegend, cat#426803) at RT for 20 minutes. The cells were then washed with cyto-Fast Perm Wash solution (BioLegend, cat#426803) and incubated with fluorochrome-conjugated antibodies specific to intracellular proteins for 25 minutes at RT. Following incubation, the cells were washed twice with cyto-Fast Perm buffer, resuspended in cell staining buffer, and acquired on a flow cytometer. Antibody specificity was verified by staining with respective isotype control antibodies and fluorescence-minus-one (FMO) controls. Freshly prepared samples or those analyzed within 24 hours were acquired using the LSR Fortessa X50 flow cytometer (BD Biosciences). Data analysis was performed using FlowJo v10.8.1 software, as previously described [7].

**Antibodies used for flow cytometry:** The following fluorescent dye-conjugated antibodies, purchased from BioLegend, CA, were used for flow cytometry. For Human Samples: APC anti-human CD276 (clone MIH42, cat#351006), PE anti-human Ganglioside GD2 (clone 14G2a, cat#357304), APC anti-human/mouse granzyme B (clone QA16A02, cat#372204), PE/Dazzle 594 anti-human perforin (clone dG9, cat#308132), Alexa Fluor 700 anti-human CD3 (clone OKT3, cat#317340), PE anti-human CD16 (clone 3G8, cat#302008), APC/FireTM 750 anti-human CD56 (NCAM) (clone 5.1H11, cat#362554), PE anti-human CD8 (clone SK1, cat#344706), APC anti-human CD107a (clone H4A3, cat#328620). For Mouse Samples: Brilliant Violet 421 anti-mouse/human ki-67 (clone 11F6, cat#151208), APC/FireTM 750 anti-mouse NK1.1 (clone PK136, cat#108752), Alexa Fluor 700 anti-mouse CD3 (clone 500A2, cat#152316), PE anti-mouse perforin (clone S16009A, cat#154306), Alexa Fluor 594 anti-mouse CD45 (clone 30-F11, cat#103144), PE anti-mouse CD8a (clone 53-6.7, cat#100708).

**Enzyme-linked immunosorbent assay (ELISA).** ELISA was performed to measure the concentrations of TNF-α and IL-2 in the culture supernatants. ELISA MAX™ Standard Set Human TNF-α (cat#430201) and ELISA MAX™ Standard Set Human IL-2 (cat#431801) from Biolegend were used, following the manufacturer's instructions. Briefly, 96-well Nunc MaxiSorp ELISA plates (Biolegend, cat#423501) were coated with capture antibody (100 µl/well) overnight at 4°C. The plates were washed four times with wash buffer (PBS + 0.05% tween-20) and then blocked with assay diluent (200 µl 10% FBS in PBS/well) for one hour at RT on a shaker. After washing the plates four times, the cell culture supernatants were collected, and mouse TNF-α or IL-2 standards at concentrations of 500, 250, 125, 62.5, 31.3, and 15.6 pg/mL were freshly prepared. The cell culture supernatants (diluted three times) or standards (100 µL/well) were added in triplicates to the wells and incubated at RT on a shaker for two hours. The plates were washed four times and then incubated with avidin-HRP solution (100 µL/well) at RT for 30 minutes with shaking. After washing the plates five times, tetramethylbenzidine substrate solution (100 µL/well) was added. The plates were incubated in the dark until the desired blue color developed. The reaction was stopped by adding stop solution (100 µL/well), and the absorbance was read at 450 nm and 570 nm using a plate reader. The absorbance at 570 nm was subtracted from the absorbance at 450 nm to obtain the final readings, which were then compared with standard curves. Each sample was assayed in triplicate and the experiment was repeated more than three times.

**The NK cell-mediated cytotoxicity assay by luminescence**. Luciferase gene-expressing stable SK-N-B(E)2-C cells were used for the assay. The cells were seeded in a 12-well plate ($1x10^5$/well) cells in RPMI medium containing 10% FCS (fetal calf serum) for 24 hours. The cells were transfected with miRNAs (60 nM) using lipofectamine RNAiMAX (ThermoFisher Scientific, cat#13778150) transfection reagent and Opti-MEM reduced serum transfection medium (ThermoFisher Scientific, cat#31985070) for 9 hours, followed by replacement with fresh RPMI medium containing 2% FCS. After 48 hours, activated NK cells (effector [E]) were cocultured with SK-N-B(E)2-C (target [T]) cells at a 1:1 E and T ratio for 6 hours. After the coculture, cells were collected, washed with PBS, and lysed in 100 µl of reporter lysis buffer (Promega, cat#E3971). One freeze-thaw cycle was performed to ensure complete cell lysis. The cell lysates were centrifuged, and the cleared lysates (80 µL) were transferred to a white, flat-bottom 96-well plate. The substrate D-luciferin (Promega Corporation, #E151A) was added (80 µL/well) and Luminescence was measured using a luminescence plate reader (Infinite 200 PRO, Tecan). The

results were presented as the percentage of relative luminescence units, indicating the level of cytotoxicity.

**Fluorescence microscopy.** The NK cell-mediated cytotoxicity assay was carried out using fluorescence microscopy. Following the coculture of NB cells with NK cells, the NK cells were removed, and the NB cells were gently washed with PBS. The tumor cell survival was determined by counting the number of GFP fluorescence-positive cells using fluorescence microscopy.

**Immunohistochemistry (IHC) staining.** The IHC staining was performed on a Discovery Ultra advanced staining system (Roche Diagnostics, Ventana Medical Systems, Inc.) through our institutional core facility. In brief, the tumor sections or PDX tissues were fixed in 70% ethanol, embedded in paraffin, and cut into 5-mm sections. The sections were deparaffinized in a mild detergent solution and mixed by vortexing at 69°C for 24 min (Roche Diagnostics, cat#950-102). Tris-based reaction buffer, pH 7.6 (Roche Diagnostics, cat# 950-300), was used throughout the protocol to maintain aqueous conditions and rinse slides. Antigen retrieval was performed by treatment with cell conditioning CC1, 8.2 pH tris-borate-EDTA buffer, at 95°C for 32 min (Roche Diagnostics, cat# 950-124), followed by Discovery ChromoMap RUO Inhibitor treatment for 8 min at room temperature. Next, sections were incubated with primary antibodies, including anti-human B7-H3 (Proteintech, cat# 66481), anti-mouse CD34 (clone MEC14.7, BioLegend, cat# 119302), anti-mouse F4/80 (clone BM8, BioLegend, cat# 123102), anti-mouse CD68, anti-mouse Ki67, cleaved caspase-3 (Asp175) (Cell Signaling Technology, cat# 9661), and anti-mouse CD161c/NK1.1 polyclonal antibody (Bioss antibodies, cat# bs-4682R), at 37°C for 32 min within the Discovery Benchmark ULTRA advanced staining system. The HRP-conjugated secondary antibody Discovery anti-rabbit HQ RTU (Roche Diagnostics, cat# 760-4815) was performed for 16 min at 37°C. After that, samples were incubated with enzyme-conjugated biotin-free Discovery anti-HQ horse-radish peroxidase RTU (Roche Diagnostics, cat# 760-4820) at 37°C for 16 min, followed by chromogen staining with Discovery ChromoMap DAB RUO and counterstaining with Hematoxylin II (Roche Diagnostics, cat# 760-159, 790-2208 & 760-2037) for microscopic examination. The images were acquired using a fluorescence microscope (Leica Microsystems Inc, USA). Tumor microvessel density was assessed based on CD34-positive staining and accessed according to the criteria described by Weidner and colleagues [10, 11]. Firstly, the entire tumor section was scanned at low magnification to find areas with high vessel density. Then, images of four such fields were digitally acquired at high magnification (20X). Microvessel density was quantified by manual counting using Photoshop (version 22.0.1), and an average of four fields from each tumor was determined. Five mice from each group were included in the analysis.

**Cell counting by trypan blue exclusion method.** We assessed the impact of microRNAs on NB cell survival over time through a trypan blue exclusion method. NB cells were mixed with 0.1 mL of trypan blue stock solution (0.4% solution in PBS) at a 1:1 ratio (0.1 mL of cells + 0.1 mL of trypan blue) to enable cell staining. The trypan blue-cell mixture was loaded onto a hemacytometer. The cells were immediately examined under a microscope at low magnification. Manual counting was performed to determine the number of cells that exhibited blue staining (non-viable cells) and the total number of cells present. Cell viability was calculated by dividing the number of viable cells (non-stained cells) by the total number of cells counted within the grids on the hemacytometer. For each biological replicate, three aliquots from each sample were counted separately.

**Colony formation assay**. The colony formation assay was performed to investigate the impact of microRNAs on the tumor-inhibiting capabilities of NB cells to form colonies and survive over time. In this in vitro assay, the ability of a single adherent cell to survive and expand into a clonal population over time was assessed. To conduct the assay, NB cells were seeded at very low densities in a 6-well plate, with 1000 cells per well in 6 ml of growth medium. The cells were then incubated for a period of 2 weeks to allow colony formation. After the incubation period, the colonies were fixed with methanol to immobilize the cells and preserve their morphology. Subsequently, the colonies were stained with 0.5% crystal violet, which made them visible and distinguishable. To quantify the colony formation, the stained colonies were manually counted using the count tool in Photoshop (version 22.0.1). By counting the colonies, cell survival curves were plotted and quantified the data.

**Wound healing assay.** NB cells were seeded into a 12-well tissue culture plate at a density that allowed them to reach 80% confluence after 24 hours of growth. The next day, cells were transfected with microRNAs for 8 hours, as described above. On the following day, a wound was created by scraping the cell layer in a straight line using a 200 µl sterile pipette tip. After the scratch, cells were gently washed with PBS to remove detached cells and replenished with fresh growth medium. Cells were then photographed using a phase contrast microscope at 10x magnification. After 20-36 hours, based on the cell line, images were taken again to observe cell migration. The wound width was calculated in pixels using digital imaging (Photoshop).

**Neurosphere-formation Assay**. To evaluate the impact of miRNAs on the self-renewal and tumor aggressiveness properties of NB cells, an in vitro neurosphere-formation assay was conducted. NB cells were plated at a density of 20,000 cells per well in a 12-well ultra-low attachment plate. The cells were cultured in neural stem cell serum-free DMEM/F12 medium, which lacks an adherent substrate but contains essential growth factors including EGF (20 ng/mL), bFGF (40 ng/mL), Heparin (2 µg/mL), β-Mercaptoethanol (0.1 mM), B27 supplement (1%), N2 supplement (1%), and Pen-Strep (1%). The cells were allowed to grow and form neurospheres over a 4-day period. The neurospheres formed in the cultures were monitored using a phase-contrast microscope equipped with a camera. The average size of the neurospheres was calculated in pixels using Photoshop and quantified.

**Cytokine arrays.** Cytokine analysis was conducted using the Proteome Profiler Mouse XL Cytokine Array (cat# ARY028 from R&D Systems), following the manufacturer's guidelines. A total of 200 µg of total protein was utilized. Arbitrary values representing cytokine abundance were determined based on spot pixel densities within each dot plot and were subsequently normalized against reference spots. These measurements were acquired and processed using Image Studio Lite software from LI-COR Biosciences.

**NB mouse xenografts**. The impact of miRNAs on tumorigenicity and immunophenotyping was investigated in two mouse xenograft models derived from cell lines. All experimental protocols were approved by the Institutional Animal Care and Use Committee at UNMC. Female C57BL/6 mice aged 5-6 weeks were obtained from The Jackson Laboratory (Bar Harbor, ME) for the experiments. After one week of adaptive feeding, the mice were divided randomly. Murine NB cell lines 9464D and NB975 were generated, which were stably engineered to express miR-29a,

miR-29b, and miR-29c or non-targeting miR controls. For the xenograft experiments, 9464D cells (2 x$10^6$) or NB975 cells (3 x$10^6$) were suspended in a mixture of 100 µl Matrigel (Corning Corporation, #CB-40230C) and PBS. The cell mixtures were then subcutaneously injected into the flank of C57BL/6 mice. Daily monitoring was conducted to ensure the health of the injection sites. After 30 days, the mice were sacrificed, and the tumors were excised. Photographs were taken, and the tumor weight was measured. Tumor volumes were calculated using the formula: V = LW2/2, where L represents tumor length and W represents tumor width. The sample size is described in the corresponding figure legends. The investigator conducted the experiment while blinded to the group allocation of the mice. For immunophenotyping analysis, the tumors were minced using scissors and incubated with tumor digestion buffer, which consisted of a mixture of 5 ml stock (500 µL Liberase DL Research Grade from Roche, cat# 05401160001), 750 µL of 1 mg/ml DNase I solution, and 3.75 ml RPMI 1640 medium. The minced tumors were incubated in this buffer for 30 minutes at 37°C and then filtered through 70-mm cell strainers. The resulting single-cell suspension was processed for immunophenotyping analysis using flow cytometry, following a protocol previously described [7].

**Statistical analysis**. The data is presented as mean ± SEM (standard error of the mean). The data was derived from 3-4 independent experiments or independent biological replicates. The statistical significance of the results was determined using the two-tailed Student t-test. For survival analysis, the Kaplan-Meier method was used to estimate and visualize the survival rates over a specified time period. The log-rank test was then employed to evaluate the statistical significance of any differences in survival between groups. A significance level of $p < 0.05$ was considered statistically significant.

**Code availability:** The code is available upon request.

## SUPPLEMENTARY REFERENCES


1 Molenaar JJ, Koster J, Zwijnenburg DA, van Sluis P, Valentijn LJ, van der Ploeg I *et al*. Sequencing of neuroblastoma identifies chromothripsis and defects in neuritogenesis genes. *Nature* 2012; 483: 589-593.

2 Rajbhandari P, Lopez G, Capdevila C, Salvatori B, Yu J, Rodriguez-Barrueco R *et al*. Cross-Cohort Analysis Identifies a TEAD4-MYCN Positive Feedback Loop as the Core Regulatory Element of High-Risk Neuroblastoma. *Cancer Discov* 2018; 8: 582-599.

3 Misiak D, Hagemann S, Bell JL, Busch B, Lederer M, Bley N *et al*. The MicroRNA Landscape of MYCN-Amplified Neuroblastoma. *Front Oncol* 2021; 11: 647737.

4 Kocak H, Ackermann S, Hero B, Kahlert Y, Oberthuer A, Juraeva D *et al*. Hox-C9 activates the intrinsic pathway of apoptosis and is associated with spontaneous regression in neuroblastoma. *Cell Death Dis* 2013; 4: e586.

5 Rifatbegovic F, Frech C, Abbasi MR, Taschner-Mandl S, Weiss T, Schmidt WM *et al*. Neuroblastoma cells undergo transcriptomic alterations upon dissemination into the bone marrow and subsequent tumor progression. *Int J Cancer* 2018; 142: 297-307.

6 Lambertz I, Kumps C, Claeys S, Lindner S, Beckers A, Janssens E *et al*. Upregulation of MAPK Negative Feedback Regulators and RET in Mutant ALK Neuroblastoma: Implications for Targeted Treatment. *Clin Cancer Res* 2015; 21: 3327-3339.

7 Pathania AS, Prathipati P, Olwenyi OA, Chava S, Smith OV, Gupta SC *et al*. miR-15a and miR-15b modulate natural killer and CD8(+)T-cell activation and anti-tumor immune response by targeting PD-L1 in neuroblastoma. *Mol Ther Oncolytics* 2022; 25: 308-329.

8 Pathania AS, Wani ZA, Guru SK, Kumar S, Bhushan S, Korkaya H *et al*. The anti-angiogenic and cytotoxic effects of the boswellic acid analog BA145 are potentiated by autophagy inhibitors. *Molecular cancer* 2015; 14: 6.

9 Livak KJ, Schmittgen TD. Analysis of relative gene expression data using real-time quantitative PCR and the 2(-Delta Delta C(T)) Method. *Methods* 2001; 25: 402-408.

10 Weidner N, Semple JP, Welch WR, Folkman J. Tumor angiogenesis and metastasis--correlation in invasive breast carcinoma. *N Engl J Med* 1991; 324: 1-8.

11 Weidner N, Folkman J, Pozza F, Bevilacqua P, Allred EN, Moore DH *et al*. Tumor angiogenesis: a new significant and independent prognostic indicator in early-stage breast carcinoma. *J Natl Cancer Inst* 1992; 84: 1875-1887.
