# Supplementary material for: The miR-29 family facilitates the activation of NK-cell immune responses by targeting the B7-H3 immune checkpoint in neuroblastoma
Source: Cell Death Dis. 2024 Jun 18;15(6):428. doi: 10.1038/s41419-024-06791-7 (PMC11189583; doi:10.1038/s41419-024-06791-7)

Uncropped blots

Fig. 1H

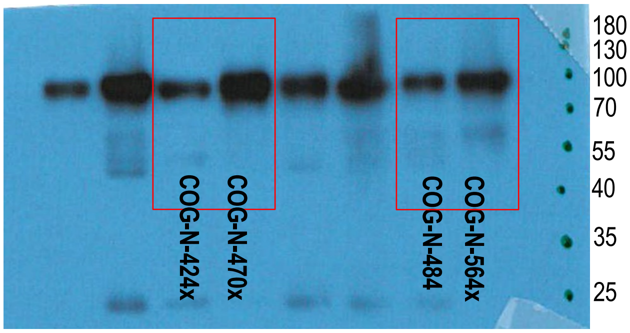

B7-H3 (MW ~100 KD)

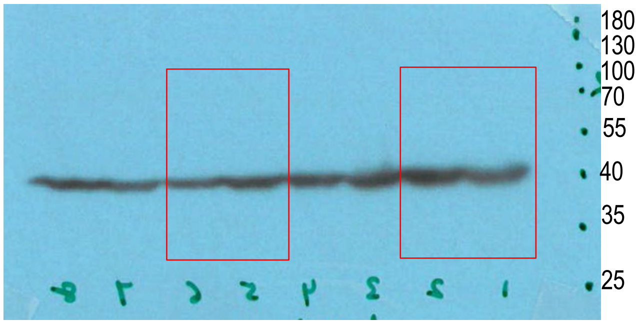

β-actin (MW ~44 KD)

Figure 3A

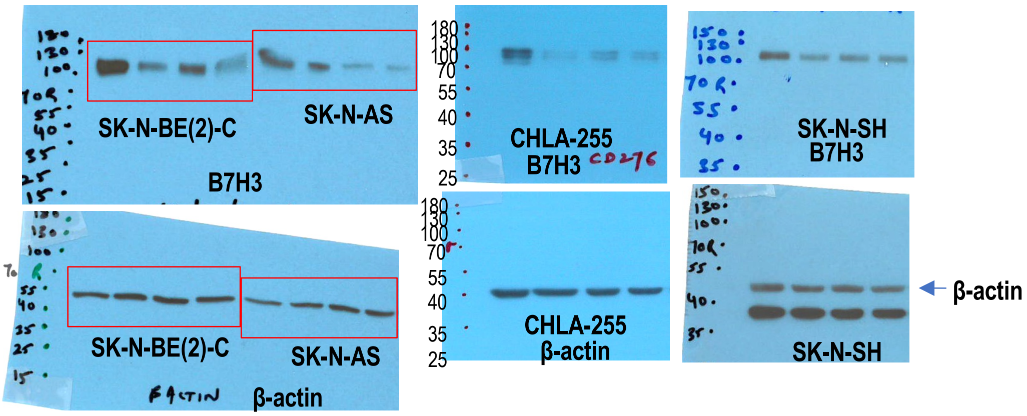

Figure 3D

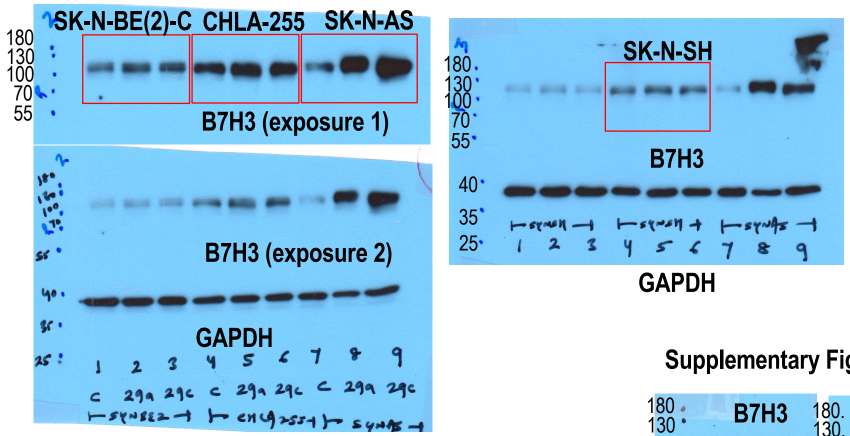

Supplementary Figure 3H

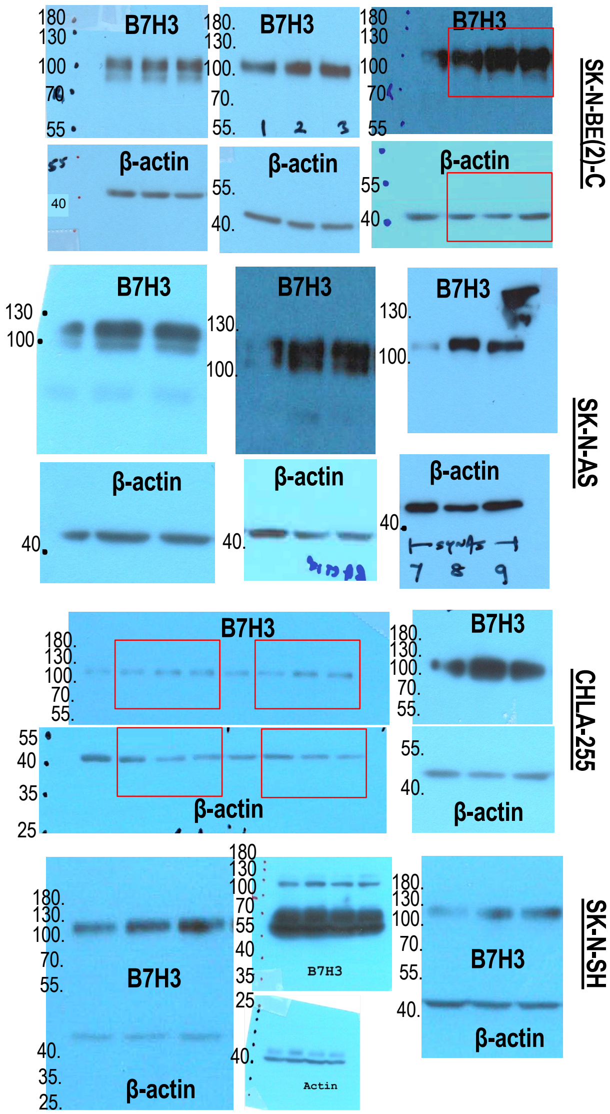

Supplementary Figure 3A

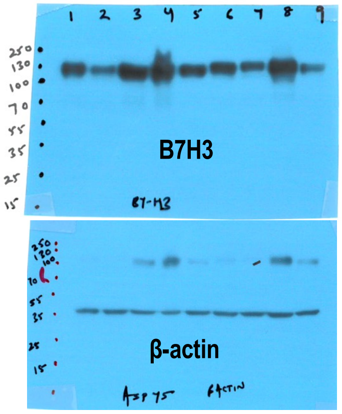

Supplementary Figure 3E

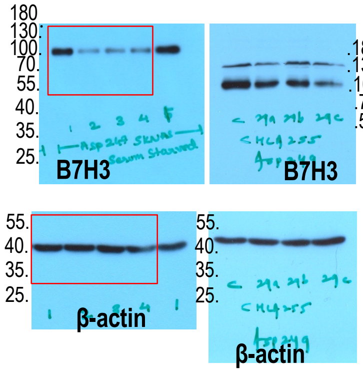

Supplementary Figure 7E raw blots

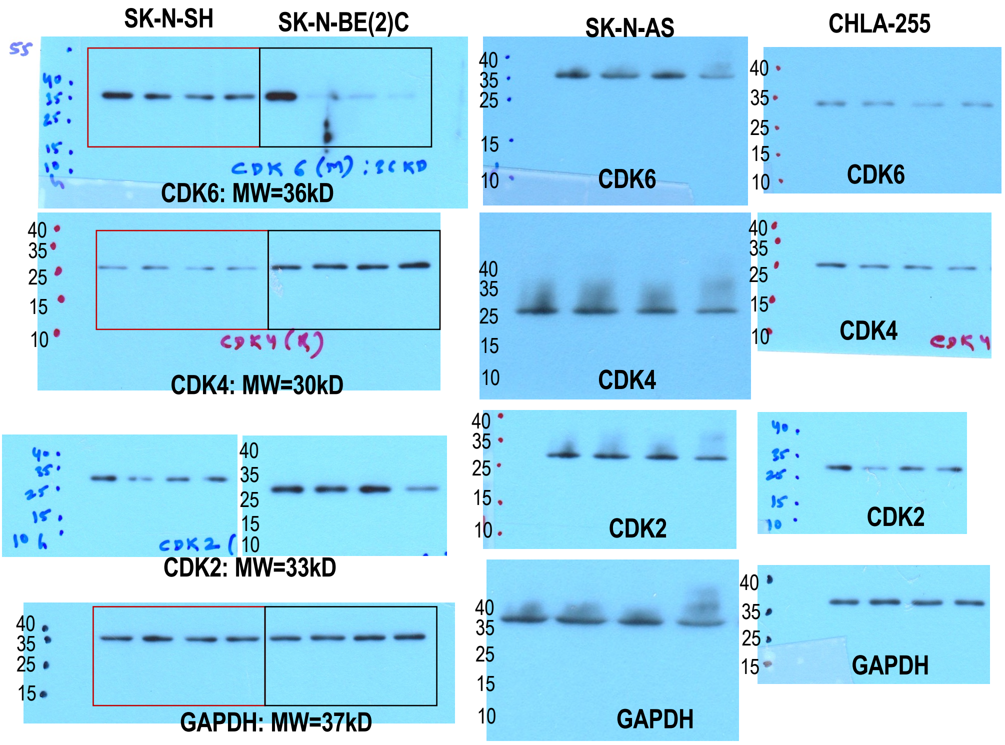

Supplement: Supplementary file 2 — Uncropped images [file 41419_2024_6791_MOESM2_ESM.pdf]
